# Metabolic Heterogeneity of Brain Tumor Cells of Proneural and Mesenchymal Origin

**DOI:** 10.3390/ijms231911629

**Published:** 2022-10-01

**Authors:** Corinna Seliger, Anne-Louise Meyer, Verena Leidgens, Lisa Rauer, Sylvia Moeckel, Birgit Jachnik, Judith Proske, Katja Dettmer, Tanja Rothhammer-Hampl, Leon D. Kaulen, Markus J. Riemenschneider, Peter J. Oefner, Marina Kreutz, Nils-Ole Schmidt, Marsha Merrill, Martin Uhl, Kathrin Renner, Arabel Vollmann-Zwerenz, Martin Proescholdt, Peter Hau

**Affiliations:** 1 Department of Neurology and Wilhelm Sander-NeuroOncology Unit, University Hospital Regensburg, 93053 Regensburg, Germany; 2 Department of Neurology, University Hospital Heidelberg, 69120 Heidelberg, Germany; 3 Department of Psychosomatic Medicine and Psychotherapy, University Medical Center Freiburg, 79104 Freiburg, Germany; 4 Institute of Functional Genomics, University of Regensburg, 93053 Regensburg, Germany; 5 Department of Neuropathology, Regensburg University Hospital, 93053 Regensburg, Germany; 6 Department of Internal Medicine III, University Hospital Regensburg, 93053 Regensburg, Germany; 7 Department of Neurosurgery, University Hospital Regensburg, 93053 Regensburg, Germany; 8 Surgical Neurology Branch, National Institute of Neurological Diseases and Stroke, Bethesda, MD 20892, USA; 9 Department of Neurology, University Hospital Erlangen, 91054 Erlangen, Germany

**Keywords:** glioma, metabolism, metformin

## Abstract

Brain-tumor-initiating cells (BTICs) of proneural and mesenchymal origin contribute to the highly malignant phenotype of glioblastoma (GB) and resistance to current therapies. BTICs of different subtypes were challenged with oxidative phosphorylation (OXPHOS) inhibition with metformin to assess the differential effects of metabolic intervention on key resistance features. Whereas mesenchymal BTICs varied according to their invasiveness, they were in general more glycolytic and less responsive to metformin. Proneural BTICs were less invasive, catabolized glucose more via the pentose phosphate pathway, and responded better to metformin. Targeting glycolysis may be a promising approach to inhibit tumor cells of mesenchymal origin, whereas proneural cells are more responsive to OXPHOS inhibition. Future clinical trials exploring metabolic interventions should account for metabolic heterogeneity of brain tumors.

## 1. Introduction

Glioblastoma (GB) is the most common and malignant primary brain tumor. Median overall survival of patients with GB ranges from 15 to 21 months [1,2]. Resistance to current therapy is thought at least in part to be driven by brain-tumor-initiating cells (BTICs) [3]. BTICs can self-renew and differentiate into several cell lineages [3]. GBs are characterized by a significant inter- and intratumoral heterogeneity [4]. A number of molecular classification systems exist, with the classical, proneural, and mesenchymal subtype being the most prevalent ones [5,6,7]. Mesenchymal BTICs are thought to be more invasive than proneural BTICs [8]. So far, the reasons for a differential invasiveness of specific tumor cells have not been understood conclusively. As invading tumor cells require significant amounts of energy [9] and use extracellular acidification and proteolytic degradation for their infiltration of healthy brain [10], we have hypothesized that tumor metabolism may play a key role in their differential functional behavior.

We performed several studies with approved drugs that target tumor metabolism including metformin [11,12]. Metformin is an approved drug for the treatment of type 2 diabetes and a synthetic derivate of galegine/guanidine, which was originally found in the traditional herbal medicine *Galega officinalis* (also known as goat’s rue) [13]. In line with differences in invasion capacity among BTIC subtypes, we observed different responses to metabolic drugs [12]. Metformin was found to alter AMPK and mTOR signaling in tumor cells by inhibition of oxidative phosphorylation [14]. It has thereby attracted great scientific interest as a potential anti-cancer drug. Derived from epidemiological data [15,16,17,18] and supported by *in vitro* and *in vivo* analyses [11,12,19,20], metformin is currently in the focus of several clinical trials with glioma patients (NCT01430351, NCT02496741, NCT02149459, NCT02780024, NCT03243851, NCT03151772, NCT01528046, and NCT02201381).

As we observed significant differences in invasion capacity among BTIC subtypes and variable responses to metabolic drugs, we hypothesized that subtype-specific metabolic phenotypes may drive tumor cell malignancy. We propose that a better understanding of specific metabolic vulnerabilities of BTICs may spur the development of novel therapeutic strategies. 

## 2. Results

### 2.1. Characteristics of Proneural and Mesenchymal BTICs and TCs

**Figure 1:**
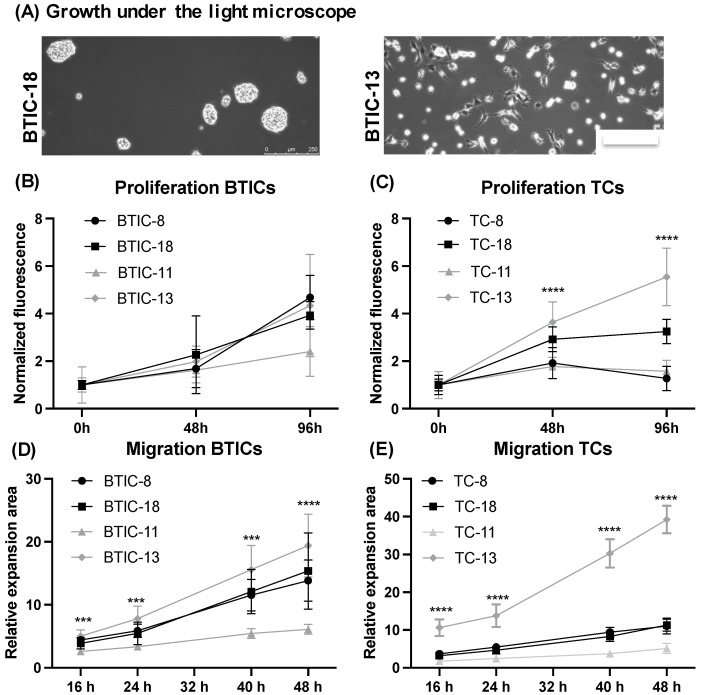
Characteristics of proneural and mesenchymal BTICs and TCs. (**A**) Proneural BTIC as exemplarily shown for BTIC-18, grew in spheres or semi-adherent, whereas mesenchymal BTICs, as exemplarily depicted for BTIC-13, showed adherent growth under the light microscope. (**B**) Proliferation assays were performed using the CyQuant Direct Cell Proliferation Assay and normalized to the 0 h value. Proliferation of proneural BTIC-8 and -18 and mesenchymal BTIC-11 and -13 varied across subtypes, which could also be observed for the corresponding differentiated TCs (**C**). Migration of BTICs (**D**) and TCs (**E**) in vitro was assessed using spheroid migration assays and normalized to the 0 h value. Again, migratory capacities varied across subtypes and differentiation states. All assays were performed in triplicate. Asterisks indicate *** *p* < 0.001, and **** *p* < 0.0001.

We analyzed four different primary BTIC lines and their respective differentiated tumor cell (TC) counterparts (Supplementary Table S1) as our core set of cells. Based on microarray analyses, two BTIC lines each were classified as proneural (BTIC-8, BTIC-18) and mesenchymal (BTIC-11, BTIC-13), respectively, according to the Verhaak classification [5]. Classification into molecular subgroups was confirmed using next generation sequencing (Supplementary Figure S1). All BTICs were IDH wildtype in culture. Proneural BTICs grew in spheres or semi-adherent, whereas mesenchymal BTICs grew adherent or semi-adherent (Supplementary Table S1, Figure 1A). 

Proliferation of BTICs and TCs varied across different subtypes and differentiation states (Figure 1B,C). In spheroid migration assays, mesenchymal BTIC-13 (Figure 1D) and TC-13 (Figure 1E) migrated farther than proneural BTICs and TCs (−8, −18), but also compared to mesenchymal BTIC-11 and TC-11. 

As conclusions on subtype-specific migratory behavior based on two lines per group may not be possible, we performed further spheroid migration assays with an extended set of cells lines, as previously described [21]. Among eight BTICs and eight TCs of proneural and mesenchymal origin, there were no significant differences between subtype-specific groups, although mesenchymal BTICs and TCs showed a trend towards increased migration as compared to proneural cells (Supplementary Figure S2). Similarly, there were no significant differences in proliferative capacity among 8 BTICs and 8 TCs of proneural and mesenchymal origin (Supplementary Figure S2).

We verified our results in situ by measuring invasion on organotypic brain tumor slices (OBSCs) for exemplary lines. Thereby, green-fluorescent mesenchymal and red-fluorescent proneural BTICs and TCs were monitored over 14 days under the fluorescent microscope (Supplementary Figure S2I–K). Corresponding to results from spheroid migration assays, mesenchymal BTIC-13 and TC-13 showed high-, and proneural BTIC-18 and TC-18 low-invasive capacity. 

### 2.2. Response of Proneural and Mesenchymal BTICs and TCs to OXPHOS Inhibition

Previous assays had already indicated significant heterogeneity between BTICs and TCs treated with metabolic agents [12]. To explore this observation further, we assessed the response of several BTICs and TCs to metformin, which was used as a model substance to inhibit OXPHOS. 

**Figure 2:**
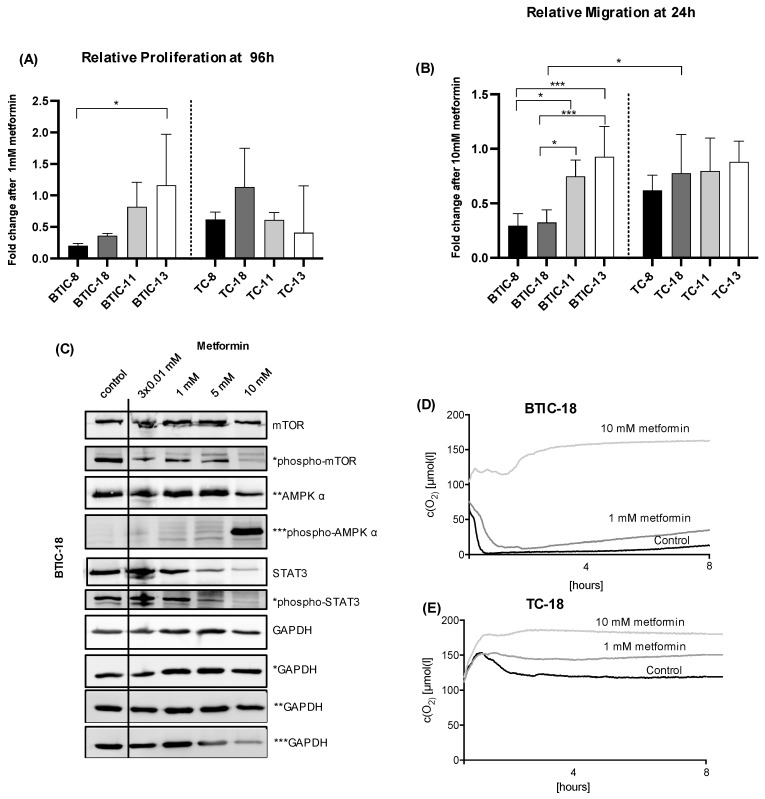
Effects of metformin on BTICs and TCs. (**A**) We compared proliferation of proneural (-8, -18) and mesenchymal BTICs (-11, -13) and TCs with or without 1 mM metformin after 96 h of treatment (fold change metformin/control). Proliferation was normalized to the 0 h value. Proneural BTIC-8 and -18 showed lower proliferation rates after metformin treatment than mesenchymal BTIC-11 and -13. (**B**) Migration of proneural (-8, -18) and mesenchymal (-11, -13) BTICs and TCs with or without 10 mM metformin was measured after 24 h of treatment. Migration was normalized to the 0 h value and results are presented as fold change metformin/control. Proneural BTIC-8 and -18 showed lower migration rates than mesenchymal BTIC-11 and 13, and also compared to proneural and mesenchymal TCs. (**C**) Increasing doses of metformin led to inhibition of mTOR and activation of AMPK in Western blot analyses after 48 h of treatment in BTIC-18. Asterisks next to the blots are indicative of the corresponding GAPDH control. Oxygen consumption over 8 h (PreSens Assay) decreased under treatment with metformin in both (**D**) BTIC-18 and (**E**) TC-18, but levels of endogenous oxygen consumption varied markedly. All assays were performed in triplicate. Significant results are only depicted for valid comparisons, i.e., comparing proneural and mesenchymal BTICs or proneural and mesenchymal TCs, or the corresponding BTIC and TC pair. Asterisks indicate * *p* < 0.05, ** *p* < 0.01, *** *p* < 0.001.

The effects of metformin on proliferation were explored using the CyQuant Direct Cell Proliferation Assay 48 and 96 h after treatment with various concentrations of metformin. Treatment with metformin led to a dose- and time-dependent inhibition of proliferation among the four investigated BTICs and four TCs (Figure 2A and Figure S3). After 96 h of high-dose metformin treatment, the fluorescence signal implicating cell number was even lower than at 0 h, which indicates cell death induction by metformin as described [22].

Proneural BTIC-8 and -18 were slightly more responsive to metformin than mesenchymal BTICs and TCs, especially at lower concentrations of metformin (Figure 2A and Figure S3). Results from the CyQuant Direct Cell Proliferation Assay could be confirmed by cell count (Supplementary Figure S3 and previously shown [12]).

The impact of increasing doses of metformin on migration was investigated in spheroid migration assays over 16, 24, 40, and 48 h in all four BTICs and four TCs (Figure 2B). Proneural BTIC-8 and -18 were significantly more responsive to 10 mM metformin after 24 h of treatment. Early time points were examined to distinguish migration from proliferation. Whereas high-, but not low-dose metformin inhibited migration of most BTICs and TCs, one mesenchymal cell line (BTIC-13) did not change its migration behavior regardless of the dose of metformin and time point (Supplementary Figure S4). 

To explore potential subtype-specific responses to metformin, we investigated proliferation and migration in an extended set of eight BTICs and eight TCs (Supplementary Figures S3–S5). There, proneural BTICs were significantly more responsive to metformin than mesenchymal BTICs and TCs (Supplementary Figure S5). 

We observed a dose-dependent activation of AMPK and inhibition of mTOR and STAT3, respectively, in proneural and mesenchymal cell lines, which are known signaling pathways altered by metformin (Figure 2C and Figure S6). 

Treatment with metformin led to a dose-dependent inhibition of cellular oxygen consumption, but basal levels of oxygen consumption differed markedly between the lines as shown for BTIC-18 (Figure 2D) and TC-18 (Figure 2E). The 10 mM metformin dose was used as a positive control, whereas lower doses of metformin were chosen to be closer to the clinically achievable doses with standard dose metformin. 

### 2.3. Oxygen Consumption of Proneural and Mesenchymal BTICs and TCs

**Figure 3:**
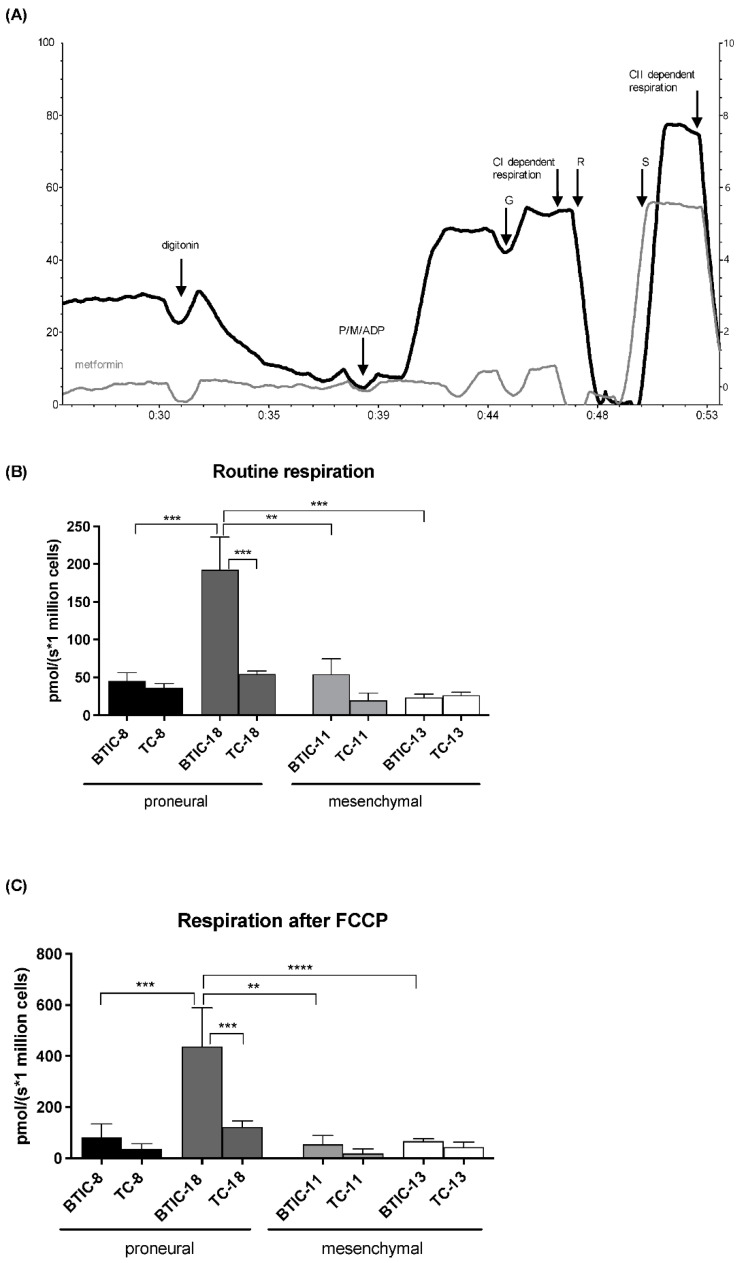
(**A**) Metformin (1 mM) inhibits complex-I-dependent respiration, but barely affects complex-II-dependent respiration in high-resolution respirometry. (**B**) Routine and (**C**) FCCP-stimulated respiration varied between BTIC and TC lines. P = pyruvate; M = malate; ADP = adenosine diphosphate; G = glutamate; CI = complex I; CII = complex II; R = rotenone; S = succinate; FCCP = carbonyl cyanide-p-trifluoromethoxyphenylhydrazone. All assays were performed in triplicate. Significant results are only depicted for valid comparisons, i.e., comparing proneural and mesenchymal BTICs or proneural and mesenchymal TCs, or the corresponding BTIC and TC pair. Asterisks indicate, ** *p* < 0.01, *** *p* < 0.001, **** *p* < 0.0001.

As we had found a differential response of proneural and mesenchymal BTICs and TCs to OXPHOS inhibition with metformin, we investigated routine and FCCP-stimulated cellular respiration among our core set of cell lines using high-resolution respirometry. In line with published data, metformin severely inhibited complex-I-dependent respiration (Figure 3A).

BTIC-18, which had shown high sensitivity to metformin in proliferation assays, respired markedly more than other BTICs at baseline and after use of the uncoupling agent FCCP. However, endogenous and FCCP-stimulated respiration showed no clear differences among other BTICs and TCs of proneural and mesenchymal origin (Figure 3B,C).

**Figure 4:**
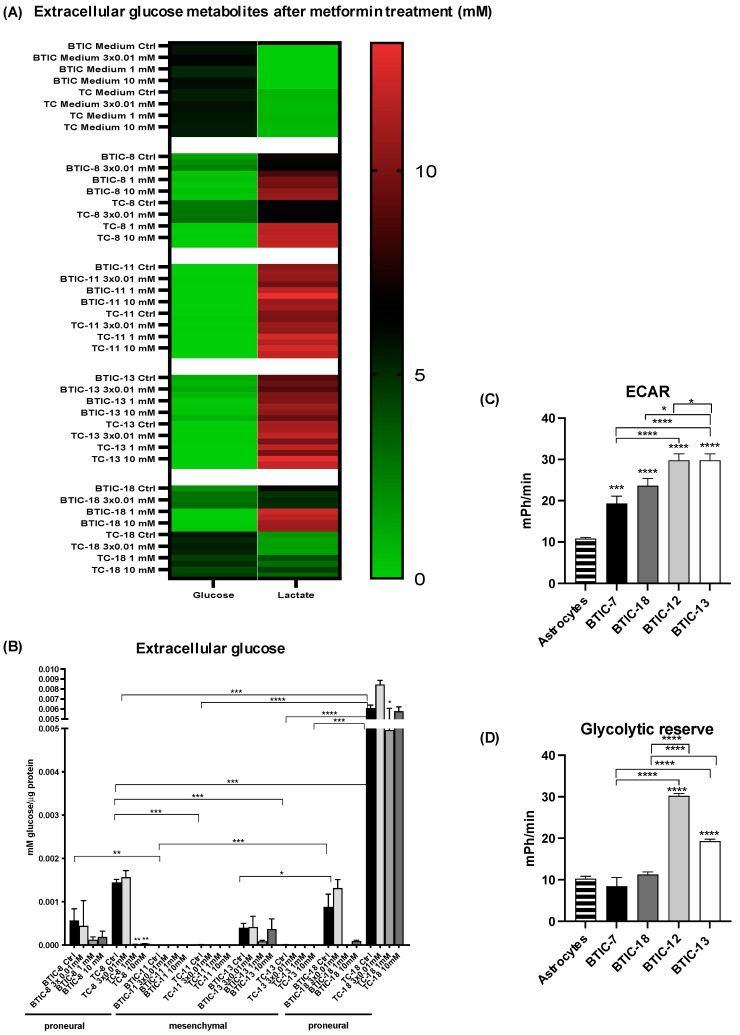
Metabolite expression and glycolytic activity. (**A**) We measured extracellular glucose and lactate after 48 h in BTIC- and TC-8, -11, -13, and -18 with or without treatment with 0.01 mM metformin three times per day (tid), 1 mM metformin and 10 mM metformin using GC/MS. (**B**) Glucose values are also depicted per µg protein. Asterisks directly above the bar indicate significance as compared to the respective control. Proneural BTIC-8 and -18 consume less glucose than mesenchymal BTIC-11. Even more pronounced, TC-8 and -18 consume far less glucose than mesenchymal TC-11 and -13. (**C**) The extracellular acidification rate and (**D**) glycolytic reserve was significantly higher in mesenchymal BTIC-12 and mesenchymal BTIC-13 than in proneural BTIC-7 or proneural BTIC-18 in Seahorse assays. Asterisks directly above the bar indicate significance as compared to astrocytes. All assays were performed in triplicate. Significant results are only depicted for valid comparisons, i.e., comparing proneural and mesenchymal BTICs or proneural and mesenchymal TCs, or the corresponding BTIC and TC pair. Asterisks indicate * *p* < 0.05, ** *p* < 0.01, *** *p* < 0.001, and **** *p* < 0.0001.

Next, we hypothesized that a differential response to OXPHOS inhibition with metformin might be due to different abilities to use or activate glycolytic rescue mechanisms. Therefore, we explored extracellular metabolites. Interestingly, mesenchymal cells consumed far more glucose than proneural cells (Figure 4A,B) and showed increased extracellular lactate levels. Treatment with metformin led to increased consumption of glucose and production of lactate. TC-18 showed the least conversion of glucose into lactate, even when metabolite levels were adjusted for protein content (Figure 4B). Within the group of mesenchymal BTICs, BTIC-11 consumed slightly more glucose than BTIC-13. 

We also performed Seahorse analyses to gain deeper insights into extracellular acidification rates (ECAR) and glycolytic reserve capacity in exemplary BTICs. The ECAR and the glycolytic reserve capacity were significantly higher in mesenchymal BTIC-12 and -13 than proneural BTIC-7 and -18 (Figure 4C,D), which may also be attributed to CO_2_ production by dehydrogenases.

### 2.4. Expression of Glycolytic Genes among Proneural and Mesenchymal Cells

**Figure 5:**
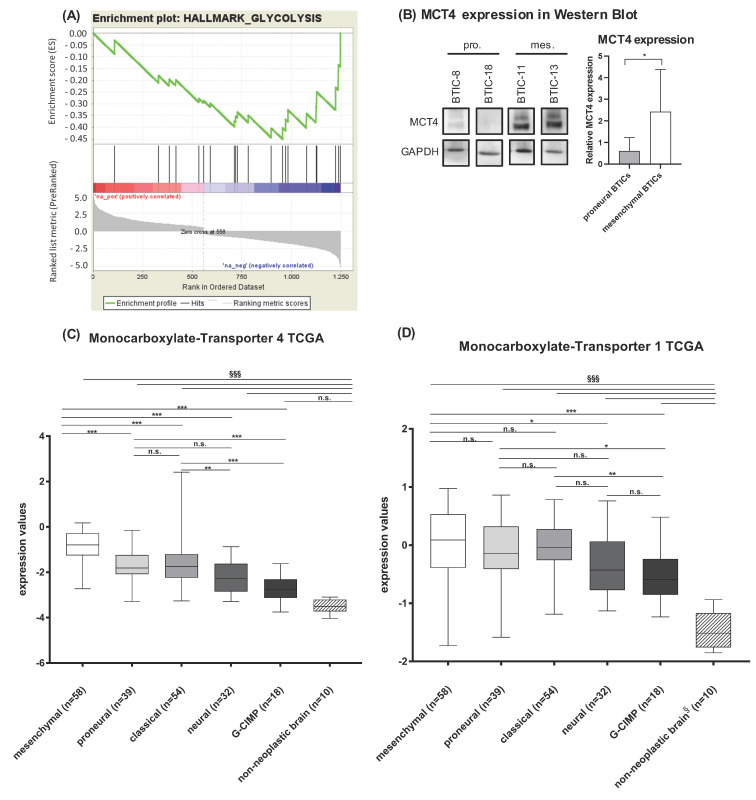
Hallmark analysis of mRNA-microarrays. (**A**) The enrichment plot indicates increased expression of glycolytic genes in mesenchymal BTICs (based on 36 lines including those used for this study). (**B**). We verified mRNA-microarray results using Western blot. Thereby, an increased protein expression of MCT4 could be confirmed, which is shown in the exemplary blot. Western blots were repeated three times with at least two biological replicates and quantified using Image J. Array and expression results were validated using TCGA-data for (**C**) *MCT4* and (**D**) *MCT1*. All assays were performed in triplicate. Asterisks indicate * *p* < 0.05, ** *p* < 0.01, *** *p* < 0.001, §§§ indicate *p* < 0.001 for comparison with non-neoplastic brain, n.s. = not significant.

To identify genetic differences underlying differential metabolic patterns of proneural and mesenchymal BTICs, we performed a gene set enrichment analysis based on the mRNA expression of 36 published BTICs including those used for the present functional and metabolic assays [23]. BTICs were segregated into proneural and mesenchymal BTICs according to the gene signatures published by Verhaak et al. [5]. The hallmark analysis showed glycolysis to differ between proneural and mesenchymal BTICs (Figure 5A), with mesenchymal BTICs showing a more glycolytic transcriptome. Significantly changed genes of the hallmark glycolysis are depicted in Supplementary Table S2, taking into consideration that microarray data may not necessarily reflect protein expression. 

Among the differentially expressed glycolytic genes, we found monocarboxylate transporter 4 (*MCT4*), which is important for the outward transport of lactate to be one interesting candidate for future research. *MCT4* mRNA expression was significantly higher in mesenchymal than proneural BTICs on both the mRNA (Supplementary Table S2) and protein level (Figure 5B) as shown for all four BTICs. Using data from the Cancer Genome Atlas (TCGA), we were able to confirm that *MCT4* (Figure 5C), but not *MCT1* expression (Figure 5D), was significantly higher in mesenchymal (n = 58) than proneural GBs (n = 39). 

To determine if MCT4 plays a major role in the response to metformin in proneural versus mesenchymal BTICs, we inhibited MCT4 in several BTICs and explored their response to metformin. Silencing of *MCT4* with siRNA was feasible (Supplementary Figure S7A–D, methods described in the Appendix A), but did not increase the inhibitory effects of metformin on tumor cell proliferation and migration (Supplementary Figure S7E,F), even after glutamine withdrawal or under hypoxia. Based on our results, although MCT4 may play a role in the different metabolic preferences of proneural and mesenchymal BTICs, sensitivity to metformin is likely based on several different genetic and metabolic alterations. 

### 2.5. Activity of Glycolytic Enzymes and Glucose Flux among Proneural and Mesenchymal BTICs

Considering that expression of glycolytic genes may not necessarily translate into increased glycolysis, we next investigated the activity of the key glycolytic enzymes hexokinase, glucose-6-phosphate dehydrogenase, and phosphofructokinase in our core set of proneural and mesenchymal BTICs. Their expression in TCGA and our lines varied according to respective isoforms with mainly hexokinase 1 and 2 being more expressed among mesenchymal BTICs (Supplementary Figure S8). 

**Figure 6:**
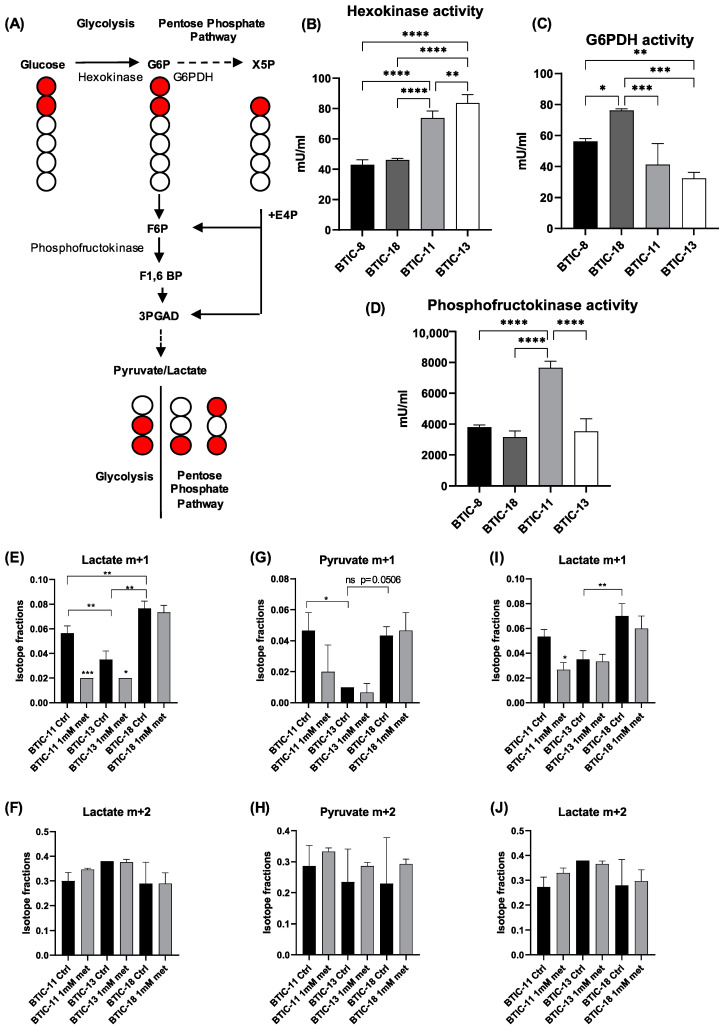
Activity of glycolytic enzymes and lactate tracing with [^13^C_2_-1,2]glucose. (**A**) Diagram depicting [^13^C_2_-1,2]glucose metabolism through glycolysis and the pentose phosphate pathway (PPP) and the resulting labeling patterns of key intermediates and lactate. Glycolysis will result in the formation of one molecule each of [^13^C_2_-2,3]lactate and [^12^C_3_]lactate from one molecule of [^13^C_2_-1,2]glucose, while the PPP will convert three molecules of [^13^C_2_-1,2]glucose into one molecule each of [^13^C_2_-1,3]lactate and [^13^C_1_-3]lactate and three molecules of [^12^C3]lactate. The relative abundances of PPP-derived [^13^C_1_-3]lactate (lactate m + 1) and glycolytic [^13^C_2_-2,3]lactate (lactate m + 2) provide estimates of PPP and glycolytic activity, respectively. Key enzymes of glycolysis and the pentose phosphate pathway are depicted in the graph. G6P = glucose 6-phosphate; X5P = xylulose 5-phosphate; G6PDH = glucose-6-phosphate dehydrogenase; E4P = erythrose 4-phosphate; F6P = fructose 6-phosphate; F1,6BP = fructose 1,6 bis-phosphate; GAP= glyceraldehyde 3-phosphate. Red filled circles denote carbon 13, black unfilled circles denote carbon 12. (**B**) Hexokinase activity was significantly lower in proneural BTIC-8 and -18 than mesenchymal BTIC-11 and -13, while (**C**) the opposite applied to G6PDH activity. (**D**) Phosphofructosekinase activity corresponded partly to hexokinase activity. (**E**) The proneural BTIC-18 contained significantly more PPP-derived [^13^C_1_-3]lactate (lactate m + 1, lactate derivative containing only C2 to C3) than the mesenchymal BTICs-11 and -13. Treatment with 1 mM metformin led to a significant drop in PPP-derived [^13^C_1_-3]lactate in BTIC-11 and BTIC-13. (**F**) The relative content of glycolytic [^13^C_2_-2,3]lactate (lactate m + 2, lactate derivative containing only C2 to C3) was increased in BTIC-13, albeit not significantly, as compared to BTIC-11 and -18. Treatment with 1 mM metformin resulted in a relative, albeit not significant increase in [^13^C_2_-2,3]lactate in BTIC-11. Utilization of [^13^C_2_-1,2]glucose via the PPP leads to the formation of (**G**) [^13^C_1_-3]pyruvate and (**I**) [^13^C_1_-3]lactate, whose m/z values increase by +1 unit in comparison to [^12^C_3_]pyruvate and [^12^C_3_-3]lactate. Utilization of [^13^C_2_-1,2]glucose via glycolysis results in double labeled (m + 2) (**H**) [^13^C_2_-2,3]pyruvate and (**J**) [^13^C_2_-2,3]lactate. Hence, a higher fraction of m + 1 pyruvate or lactate isotopologues indicates increased PPP activity. All assays were performed in triplicate. Asterisks indicate * *p* < 0.05, ** *p* < 0.01, *** *p* < 0.001, and **** *p* < 0.0001.

Mesenchymal BTICs showed increased activity of hexokinase 1 and 2, and partly of phosphofructokinase (BTIC-13), whereas proneural BTICs showed increased activity of glucose-6-phosphate dehydrogenase (Figure 6B–D). We suspected that proneural BTICs metabolized more glucose via the pentose phosphate pathway (PPP). To prove this assumption, we performed pyruvate and lactate tracing using [^13^C_2_-1,2]glucose as the tracer substrate (Figure 6A,E–J). Indeed, we were able to confirm increased flux through the PPP in BTIC-18 as indicated by the higher abundance of m + 1 isotopologues for pyruvate and lactate.

### 2.6. Relation of Key Findings to Survival Data from TCGA

We compared our results with survival data from glioma patients within the TCGA cohort. First, high expression of *MCT4* (Supplementary Figure S9A), but not *MCT1* gene transcripts (Supplementary Figure S9B) translated into inferior patient survival in the TCGA cohort. Although not reaching statistical significance, there is a trend for improved survival among patients with high expression of *G6PDH* (Supplementary Figure S9C).

## 3. Discussion

Glioblastomas are heterogeneous brain tumors [5]. In our study, we observed different invasive patterns and metabolic states of proneural and mesenchymal BTICs. In summary, mesenchymal BTICs were diverse with respect to their migratory capacity, in general more glycolytic and less responsive to OXPHOS inhibition with metformin, whereas proneural BTICs were less invasive, showed an increased flux of glucose carbons via the pentose phosphate pathway, and responded better to OXPHOS inhibition with metformin. 

In a recent study, Fayzullin et al. described a mesenchymal gene signature that was associated with increased invasion [8]. Several candidate mechanisms were described, including CD44-associated altered cell adhesion in mesenchymal cells [24]. Increased invasion is also a common phenomenon in epithelial-to-mesenchymal transition (EMT) [25] and in proneural-to-mesenchymal transition [26]. Overexpression of genes derived from a mesenchymal signature is associated with inferior survival of glioma patients [6]. It should be noted that even within a tumor, different molecular subtypes may coexist, reflecting intra-tumoral heterogeneity [27]. Further, even within subgroups the extent of invasion may vary widely, as shown in our study. A link between invasion and glycolysis has been described earlier in a different context. In that model, miRNA-451 inhibited glioma cell proliferation and invasion by downregulating glucose transporter 1 [28]. Treatment with the VEGF antibody bevacizumab led to increased tumor hypoxia, activated glycolysis, and increased brain invasion of tumor cells in an intracranial GB xenograft model [29]. Increased invasion is a consequence of activated glycolysis, as it creates an acidic environment that favors cancer compared to healthy cells [30]. Lactate, the end product of glycolysis, is generated through lactate dehydrogenase A (LDH-A), which was found to induce transcriptional regulation of matrix-metalloproteinase-2 (MMP-2) and integrin alpha(v)beta(3) in glioma cells, both facilitating migration [31]. Additionally, lactate regulates expression of TGF-beta2, whose mRNA ranked as the strongest regulated among the mesenchymal group in the hallmark glycolysis in our microarray analyses. 

Considering the easy clinical application and multilayer mode of action, we chose metformin as a model substance to study GB cell metabolism. Based on previous studies [20], we assumed that metformin’s mode of action might lead to an increased response of proneural cells due to their common mutations within metabolic pathways—one of metformin’s major targets. 

Several studies already explored effects of metformin on glioma cells. They mainly focused on effects of high-dose metformin on different functions of glioma cells including proliferation, invasion, apoptosis, and differentiation of BTICs [19,20,32]. None of those studies investigated why some cells are more responsive to metformin than others, a heterogeneity that is specifically observed in BTICs from different origins. 

For the first time, we demonstrated a specific inhibition of complex I of the respiratory chain by metformin in glioma cells using high-resolution respirometry. It should be noted, however, that there are several other targets of metformin [33]. 

Previous studies have not comprehensively explored results for distinct progenitor and differentiation states as well as molecular subgroups of brain tumor cells. It is remarkable that proneural BTICs responded to metformin with a stronger inhibition of their proliferation than TCs, similar to published results in other tumors as well as gliomas [20]. The underlying reasons may be that BTICs show elevated levels of GLUT3 glucose transporter [34], which enables them to increase their glucose uptake and, thereby, enhance their resistance to nutrient fluctuations. Furthermore, the expression of the GLUT1 transporter correlates with proliferation rates in gliomas [35]. Thus, it could be inferred that a generally enhanced proliferation of BTICs compared to TCs might result in a stronger anti-proliferative effect of metformin. 

Previous studies have not comprehensively explored the effects of metformin on molecular subgroups of brain tumor cells. Our results from transcriptome analyses, Seahorse analyses, enzyme activity assays, and analyses of extracellular metabolites found mesenchymal BTICs to be more glycolytic and less responsive to OXPHOS inhibition with metformin. Microarray data from numerous BTICs showed that glycolytic genes were differentially expressed between proneural and mesenchymal BTICs. The lactate transporter MCT4 and the glycolytic enzyme glucose-6-phosphate dehydrogenase (G6PDH) were two of the most interesting candidate genes and were hence further explored. Although a single knockdown of *MCT4* with siRNA may not be functionally relevant for BTICs, as they are able to compensate via *MCT1*, the investigation of MCT4 in glioma pathophysiology merits further research. Voss et al. (2017) described that MCT4 is enriched in areas of tumor hypoxia and that an inhibition of MCT4 binding to its chaperon basigin strongly inhibited tumor progression of glioblastoma stem cells [36]. In line with our data, Lim et al. (2014) found an upregulation of *MCT4* to correlate with an aggressive mesenchymal subset of GB [37]. Our hypothesis was further corroborated when analyzing patient data from TCGA, where high expression of *MCT4* was associated with inferior patient survival. However, survival data in the human TCGA cohort should be interpreted with caution and should be examined in conjunction with established risk factors for the disease. Combined treatment with metformin and syrosingopine, an inhibitor of MCT1/4, led to synthetic lethality via depletion of the NAD^+^ pool in cancer cells [38]. Synthetic treatment strategies may be warranted especially in mesenchymal BTICs, where metformin treatment may even exert negative effects through upregulation of *GLUT-1* leading to increased glycolysis [39] and thereby proliferation [35], which has already been described in the 1990s. 

The activity of G6PDH was higher among proneural BTICs. Cells that use mitochondrial respiration and lipolysis produce significant amounts of reactive oxygen species (ROS). For compensation of ROS, tumor cells need NADPH for glutathione regeneration. The pentose phosphate pathway generates NADPH for the reductive biosynthesis of fatty acids and antioxidant defense, as well as ribose 5-phosphate for nucleotide biosynthesis [40]. 

Our study has several limitations. Although we performed various in-depth metabolic assays with a core set of eight BTICs and TCs, additional functional assays with 16 BTICs and TCs, and array data with 36 BTICs, our sample size may have been too small to detect significant differences between BTIC subgroups, for example, regarding tumor cell migration and invasion. Furthermore, a strong heterogeneity among the investigated BTIC and TC lines makes general conclusions on tumor subtypes difficult. However, a preference of mesenchymal cells for glycolysis has already been described in a study including numerous BTIC cultures [35,41], but that study lacked in-depth metabolic analyses on their BTIC lines. Further studies should also focus on the interaction of glutamine metabolism with glucose metabolism among different GB subtypes, as GB cells may show metabolic plasticity, which may be inhibited by additional targeting of glutamine metabolism [42]. Although we explored numerous BTICs of proneural and mesenchymal origin, we were not able to compare those results to BTICs of the classical subtype due to a lack of primary lines. However, we compared data of all subtypes in TCGA analyses and other authors also found the metabolism of proneural and mesenchymal BTICs to be most decisive [41]. 

Our study has several strengths. We performed numerous in-depth metabolic assays on a variety of primary BTICs and TCs. We could show that mesenchymal BTICs were mainly glycolytic, whereas proneural BTICs were more responsive to OXPHOS inhibition with metformin. Interestingly, observational data revealed improved OS and PFS predominantly among patients with WHO grade III gliomas using metformin, indicating subgroups being more responsive to OXPHOS inhibition [17]. Lower grade gliomas and secondary glioblastomas express more frequently a proneural gene signature alongside frequent mutations within *IDH* [5]. Although based on limited experimental data [43], one clinical trial (NCT28601826) is exclusively exploring the effects of metformin on IDH-mutated solid tumors including glioma which will thereby challenge our observation of an increased susceptibility of BTICs of the proneural subtype to metformin. 

## 4. Materials and Methods

### 4.1. Cellular Models and Functional Assays

#### 4.1.1. Tumor Specimens and Enrichment of BTICs

BTICs were derived from resected, newly diagnosed human malignant gliomas as previously described [12]. The sampling of tumor specimens and enrichment of BTICs was approved by the Ethics Committee of the University of Regensburg (No° 09/101), and all patients gave written informed consent. We used the following core set of lines for our experiments: BTIC-8, BTIC-11, BTIC-13, and BTIC-18 (Supplementary Table S1). BTICs were kept in DMEM low glucose medium (DMEM with 1 g/L of glucose; Sigma-Aldrich, #D6046) containing Epidermal Growth Factor (Miltenyi Biotec, #130-097-751, Bergisch Gladbach, Germany) and Fibroblast Growth Factor (Miltenyi Biotec, #130-093-842, Bergisch Gladbach, Germany) supplemented with 50 U (*v*/*v*) Penicillin, 0.05% (*v*/*v*) Streptomycin (#P4333), 2 mM (*v*/*v*) L-Glutamine (#G7513), 1% (*v*/*v*) MEM Vitamin Solution (#M6895), and 1% (*v*/*v*) non-essential amino acids (#M7145) (all Sigma-Aldrich, St. Louis, USA). For differentiation, growth factors were withdrawn, and cells were exposed to fetal calf serum (FCS) for at least two weeks. Cells were incubated at 37 °C, 5% CO_2_, 95% humidity in a standard cell culture incubator. All experiments were performed with mycoplasma-free cells.

#### 4.1.2. Cell Proliferation and Spheroid Migration Assays 

Proliferation was assessed using the CyQUANT^®^ Direct Cell Proliferation Assay (Thermo Scientific, #C35012, Waltham, MA, USA) according to the manufacturer’s protocol. The spheroid migration assays were performed as previously described [11]. To distinguish proliferation from migration effects, we used early time points, i.e., after 16 and 24 h, which preceded the cell doubling time (average doubling time of BTICs and TCs = 45.8 h).

#### 4.1.3. Organotypic Brain Slice Cultures (OBSCs)

BTICs und TCs were lentivirally transduced using U57 pHR SFFV GFP (BTIC-13 and TC-13) and pLenti-H1-(shRNA-Neg-control)-Rsv(RFP-Bsd) plasmids (BTIC-18 and TC-18). Lentivirally transduced BTICs were grown as spheroids in agarose-coated 96-well plates (10,000 cells/well) 48 h prior to implantation. OBSC were prepared according to Gogolla et al. [44] with individual modifications as described [12]. We implanted one spheroid per slice and used three technical replicates. Spheroid diameter ranged between 3 and 8 × 10^−5^ µm^2^ depending on initial cell size. Two days were sufficient to form cell spheroids that were transferrable by pipette. The lentiviral transduction did not affect proliferation or migration compared to non-transduced counterparts.

#### 4.1.4. Treatment of BTICs with Metformin

Metformin hydrochloride (Sigma-Aldrich #PHR1084, St. Louis, MI, USA) was dissolved in DMEM low glucose (5.5 mM) medium and used at indicated doses. 

### 4.2. RNA and Protein-Based Methods

#### 4.2.1. Polymerase Chain Reaction (PCR) and Quantitative Real Time PCR (qRT-PCR)

RNA isolation and subsequent qRT-PCR isolation were performed as previously described [12]. Primer sequences are provided in the Appendix A (Table A1).

#### 4.2.2. Microarray Analysis, Clustering, and Gene Set Enrichment Analysis 

Microarray analysis was performed as described elsewhere [45]. Computational analysis was performed using R (Version 3.1.2) and Bioconductor (http://www.bioconductor.org, accessed on 11 October 2015). Genes differentially expressed between mesenchymal and proneural BTICs were identified using the package Limma [45]. We computed a ranked gene list based on logarithmic fold change expression and applied the GSEA Preranked tool of GSEA v2.2.2 (www.broadinstitute.org/gsea, accessed on 11 October 2015). Gene set collections C2, C5, and C6 from the MSigDB (www.broadinstitute.org/msigdb, accessed on 11 October 2015) were used within GSEA. Enriched gene sets with an absolute normalized enrichment score > 2 and a maximum FDR < 0.25% were considered for further evaluation. Data are deposited at the gene expression omnibus (GEO) functional genomics data repository under the accession numbers GSE51305 and GSE76990.

#### 4.2.3. RNA Sequencing to Validate Molecular Subgroups from Microarray Analysis

RNA sequencing analyses were used to determine expression subgroups after Verhaak et al. [5]. Libraries were prepared with 200–1000 ng RNA with the TrueSeq RNA-library preparation kit v2 (Illumina) according to Schulze et al. [46]. “Next generation” sequencing (NGS) was performed at a genomics core facility: Center of Excellence for Fluorescent Bioanalytics (KFB, University of Regensburg, Regensburg, Germany) on a HiSeq 1000 instrument (Illumina, San Diego, CA, USA) using the indexed, 50 cycles single read protocol and the TruSeq SBS v3 Kit (Illumina, San Diego, CA, USA). Image analysis and base calling resulted in bcl files, which were then converted into fastq files by the CASAVA 1.8.2 software (Illumina Inc., San Diego, USA). Analysis was performed using the Genomatix software (Precigen Bioinformatics Germany GmbH i.L., Munich, Germany). fastq files were mapped to the human genome assembly GRCh38 (annotation based on ElDorado 6–2015) using the Genomatix Mining Station Mapper v3.7.6.3 allowing one mismatch. All unique hits were further processed using the Genomatix Genome Analyser v3.51106, which was used to create count tables and RPKM expression values for all samples. Reads were counted locus-based, i.e., for unions of exons of genes [46]. Gene set enrichment analysis was performed with the ssGSEAProjection v9.1.1. (Genepattern, Broad Institute) with RPKM (reads per kilobase of exon model per million mapped reads) values [47]. Subtype prediction was performed with the ssGSEA module and the gene sets proposed by Verhaak RG et al. [5].

#### 4.2.4. TCGA Analysis

mRNA expression of *MCT1*, *MCT4*, and isoforms of hexokinase, glucose-6-phosphate dehydrogenase and phosphofructokinase, was analyzed using the glioblastoma dataset available at the Cancer Genome Atlas (TCGA) database [48]. 

#### 4.2.5. Western Blot

To investigate protein levels of (p)mTOR, (p)AMPK, (p)STAT3, and GAPDH, cells were harvested and lysed with RIPA buffer and subjected to Western blotting using standard protocols. Antibodies are listed in the Appendix A (Table A2). Then, 10 mM metformin were used as the positive control for activation of AMPK and inhibition of mTOR. 

### 4.3. Metabolic Assays

#### 4.3.1. Enzyme Activity Assays

The activity of hexokinase, phosphofructokinase, and glucose-6-phosphate dehydrogenase was quantified using enzymatic assays provided by Abcam (#ab136957, Cambridge, UK) according to the manufacturer’s recommendations. 

#### 4.3.2. PreSens Technology

Cellular oxygen consumption was determined non-invasively under culture conditions with PreSens technology (PreSens Precision Sensing GmbH, Regensburg, Germany) as described [11]. 

#### 4.3.3. High-Resolution Respirometry

To verify PreSens results on cellular oxygen consumption, the activity of the respiratory system was analyzed in a two-channel titration injection respirometer (Oxygraph-2k; Oroboros Instruments, Innsbruck, Austria) at 37 °C. Detailed protocols are provided in the Appendix A. 

#### 4.3.4. Extracellular Lactate and Glucose Levels

For the measurement of extracellular metabolites, a 10 µL aliquot of cell culture supernatant was dried and subjected to GC-MS analysis. Quantification was achieved using multi-point calibration curves for each analyte using the corresponding stable isotope-labeled analog as the internal standard.

#### 4.3.5. Extracellular Acidification Rate Analysis by Seahorse Technology

Tumor cells were seeded in 96-well microplates at 8000 cells per well in 80 µL growth medium. One day prior to the experiment, the sensor cartridge was placed into the utility wells containing Seahorse XF calibrant and the system was incubated overnight at 37 °C in a non-CO_2_ incubator. The next day, cells were washed in assay-specific medium and placed in the non-CO_2_ incubator for 1 h. The ports of the Seahorse cartridge were loaded with 20 µL of 80 mM glucose, 22 µL of 9 µM oligomycin, and 25 µL of 1 M 2DG (2-deoxy-D-glucose) for the glycolysis stress test. After sensor calibration, assays were run as detailed in the manufacturer’s manual by recording ECAR (extracellular acidification rate) and OCR (oxygen consumption rate). Metabolic parameters were obtained from the XF Wave software (Version 2.4.2, Agilent/Seahorse Biosciences) and calculated using Microsoft Excel.

#### 4.3.6. ^13^C-Glucose Isotope Tracing

For stable isotope tracing experiments, cells were harvested and extracts prepared as described above without the addition of internal standards. We used [^13^C_2_-1,2]glucose as the tracer substrate and measured metabolites derived therefrom. From the GC-MS full scan data, extracted ion chromatograms based on the m/z values of the individual isotopologues were used for data analysis. Stable isotope tracing data were corrected for natural abundance of ^13^C and tracer impurity using IsoCorrectoR [49]. 

The use of [^13^C_2_-1,2]glucose as the tracer substrate allows to determine the fractions of glucose catabolized via glycolysis and the pentose phosphate pathway. Single-labeled [^13^C_1_-3]pyruvate and [^13^C_1_-3]lactate (m + 1), following correction for natural abundance of ^13^C, provide an estimate of the fraction of glucose catabolized via the PPP, whereas double-labeled [^13^C_2_-2,3]pyruvate and [^13^C_2_-2,3]lactate (m + 2) derive from glycolysis. For pyruvate and lactate the [M-CH_3_]+-ions of the derivatives were analyzed. It should be noted that positional information for ^13^C incorporation is not achieved. Double-labeled pyruvate and lactate may alternatively derive from the PPP, but in that case a different labeling pattern is observed, namely [^13^C_2_-1,3]pyruvate and [^13^C_2_-1,3]lactate. Therefore, to unequivocally distinguish ^13^C carbon flow between glycolysis and the PPP, we additionally analyzed a fragment of the lactate derivative containing only C2 to C3 (*m*/*z* 117 [m + 0], 118 [m + 1], 119 [m + 2]).

### 4.4. Statistics

All statistical analyses were performed using GraphPad Prism (Version 7.05 GraphPad Software, Inc. San Diego, CA, USA), if not otherwise indicated.

We performed a two-way ANOVA to compare the results (mean values and SDs) of controls and treated BTICs. All assays were performed in triplicate. We used Tukey’s post-hoc test to control for multiple comparisons. The level of significance was set at *p* < 0.05 (asterisks indicate * *p* < 0.05, ** *p* < 0.01, *** *p* < 0.001, and **** *p* < 0.0001). Significant results are only depicted for valid comparisons, i.e., comparing proneural and mesenchymal BTICs or proneural and mesenchymal TCs, or the corresponding BTIC and TC pair. Western blots were repeated three times with at least two biological replicates and quantified using Image J, version 1.49. 

For analysis of survival data in TCGA, we divided groups into “high” and “low” expression of the respective mRNA (i.e., *MCT4*, *MCT1* and *G6PDH*) of GBs documented in TCGA using the median expression as cut-off (expression above versus below the median). Significance between groups was tested using the log-rank (Mantel–Cox) test. 

## 5. Conclusions

In summary, we observed heterogeneous metabolic preferences of proneural and mesenchymal brain-tumor-initiating cells, which varied in their response to OXPHOS inhibition with metformin. Future clinical trials exploring metabolic interventions should account for metabolic heterogeneity of brain tumors. Interventions targeting only one metabolic pathway are prone to fail, unless the tumor is suspect to specific metabolic vulnerabilities. 

## Data Availability

Microarray data are deposited at the gene expression omnibus (GEO) functional genomics data repository under the accession numbers GSE51305 and GSE76990. Further details and other data that support the findings of this study are available from the corresponding author upon request.

## Supplementary Materials

The following supporting information can be downloaded at: https://www.mdpi.com/article/10.3390/ijms231911629/s1.

## Appendix A. Primers, Antibodies and Supplementary Methods

**Table A1:** Primers.

qRT-PCR				
Gene	Forward Primer	Reverse Primer	Source	Annealing Temperature
*MCT4*	5′-CAG TTC GAG GTG CTC ATGG-3′	5′-ATG TAG ACG TGG GTC GCA TC-3′	eurofins-mwg/operon	59 °C
*HK1*	5′-GGA CTG GAC CGT CTG AAT GT-3′	5′-ACA GTT CCT TCA CCG TCT GG-3′		58 °C
*HK2*	5′-CAA AGT GAC AGT GGG TGT GG-3′	5′-GCC AGG TCC TTC ACT GTC TC-3′		58 °C
*G6PDH*	5′-ACA TGA ATG CCC TCC ACC TG-3′	5′- GGT AGT GGT CGA TGC GGT AG-3′		56 °C
*RPLPO*	5′-CTG TCT GCA GAT TGG CTA CCC-3′	5′-GAT GGA TCA GCC AAG AAG GC-3′	Genbank Accession No: NM_001002.3	56 °C

**Table A2:** Antibodies.

Western Blot (WB)	Dilution	Catalogue Number	Manufacturer
**Primary Antibodies (WB)**			
AMPKα	1:1000	#2603	Cell Signaling Technologies
p-AMPKα	1:1000	#2535	Cell Signaling Technologies
GAPDH	1:2000	#sc-48167	Santa-Cruz
MCT4	1:500	#sc-50329	Santa-Cruz
mTOR	1:1000	#2983	Cell Signaling Technologies
p-mTOR	1:1000	#5536	Cell Signaling Technologies
STAT3	1:1000	#9145	Cell Signaling Technologies
p-STAT3 (Y705)	1:1000	#9132	Cell Signaling Technologies
p-STAT3 (S727)	1:1000	#9134	Cell Signaling Technologies
**Secondary Antibodies (WB)**			
mouse anti-rabbit IgG-HRP	1:5000	#sc-2357	Santa-Cruz
goat anti-rabbit IgG-HRP	1:5000	#R-05072-500	Advansta
donkey anti-goat IgG-HRP	1:5000	#sc-2020	Santa-Cruz

**Abbreviations**: p = phosphorylated; HRP = horseradish peroxidase; WB = Western Blot.

### Appendix A.1. Supplementary Methods

#### Appendix A.1.1. Transient Transfection with siMCT4

Transient transfection of BTICs was performed using Lipofectamine RNAiMAX Reagent (#13778, Invitrogen, Carlsbad, CA, USA) with two different siRNAs directed against *MCT4* (s17416, #4390824 and siRNA 107345, #AM16708, Life Technologies, Carlsbad, CA, USA) to evaluate mRNA level changes after 24, 48, 72 and 96 h.

#### Appendix A.1.2. High-Resolution Respirometry

Cells were harvested, resuspended in culture medium and transferred to the oxygraph chambers at a final cell density of approximately 1 × 10^6^ cells/mL. In a substrate-uncoupler-inhibitor titration (SUIT) protocol, ROUTINE respiration (no additions), LEAK respiration (oligomycin-inhibited, 2 μg/mL), and the capacity of the electron transfer system (ETS) (maximum non-coupled respiration induced by stepwise (typically 2–3 steps) titration of carbonyl cyanide p-(trifluoromethoxy) phenylhydrazone (FCCP; 2 μM solved in ethanol)) were measured in intact cells respiring on substrates in culture medium.

A second protocol was applied to identify the complexes of the respiratory system that are affected by metformin. Cells were harvested, resuspended in mitochondrial respiratory medium (MiR05) and transferred to the oxygraph chambers at a final cell density of approximately 1 × 10^6^ cells/mL. After stabilization of respiration, plasma membrane was permeabilized with digitonin (1 µg/10^6^ cells) and complex I dependent respiration was stimulated by the addition of malate (2 mM), pyruvate (5 mM), glutamate (10 mM) and ADP (5 mM). Complex II related respiration was determined after inhibition of complex I with rotenone (0.5 µM) and subsequent addition of succinate (10 mM).
