# Supplementary material for: Metabolic Heterogeneity of Brain Tumor Cells of Proneural and Mesenchymal Origin
Source: Int J Mol Sci. 2022 Oct 1;23(19):11629. doi: 10.3390/ijms231911629 (PMC9569970; doi:10.3390/ijms231911629)
Supplement: Supplementary file 1 [file ijms-23-11629-s001.zip › Supplementary Figures.pdf]

(A)

Name	MA: HuGene-1_1-st-v1.na32				samples	NGS RNASeq			
	ssGSEA			heatmap		ssGSEA			heatmap
	subclass	Proneural	Mesenchymal	subclass		subclass	Proneural	Mesenchymal	subclass
BTIC-10	mesenchymal	203	4014	mesenchymal	n.d.				
BTIC-11	mesenchymal	2011	3338	mesenchymal	√	mesenchymal	11972	14502	mesenchymal
BTIC-12	mesenchymal	1331	3152	mesenchymal	√	mesenchymal	10742	15140	mesenchymal
BTIC-13	mesenchymal	933	3239	mesenchymal	√	mesenchymal	9799	13170	mesenchymal
BTIC-7	proneural	6095	-1535	proneural	√	proneural	13228	9026	proneural
BTIC-8	proneural	5239	-806	proneural	√	proneural	16104	9286	proneural
BTIC-17	proneural	5818	-592	proneural	√	proneural	13639	10674	proneural
BTIC-18	proneural	6367	-112	proneural	√	proneural	15594	10803	proneural
		>2800					>13000		

(B)

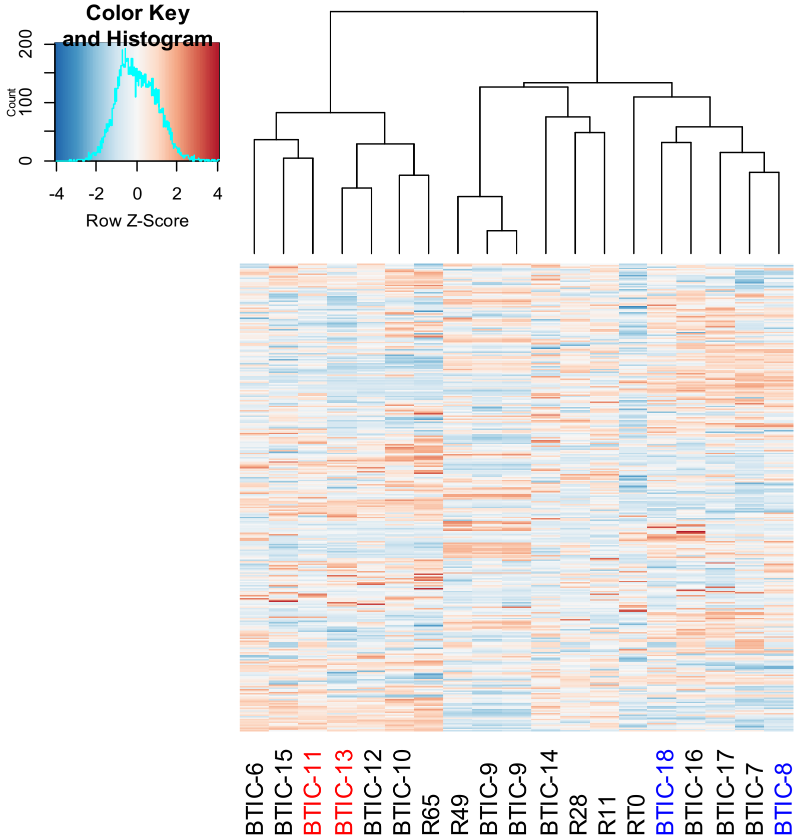

Supplementary Figure S1: RNA sequencing to validate molecular subgroups from microarray analysis. **(A)** BTIC subtyping by single sample gene set enrichment analysis (ssGSEA) using gene expression data sets generated either by microarray (HuGene-1_1-st-v1.na32) or next generation sequencing (NGS RNA seq). Subtype prediction was performed with the ssGSEA module and the gene sets proposed by Verhaak RG et al.[5] **(B)** Heatmap of the Top 1000 differentially expressed genes (sorted by adjusted p-value, based on normalized counts). Hierarchical clustering separates proneural and mesenchymal cells.

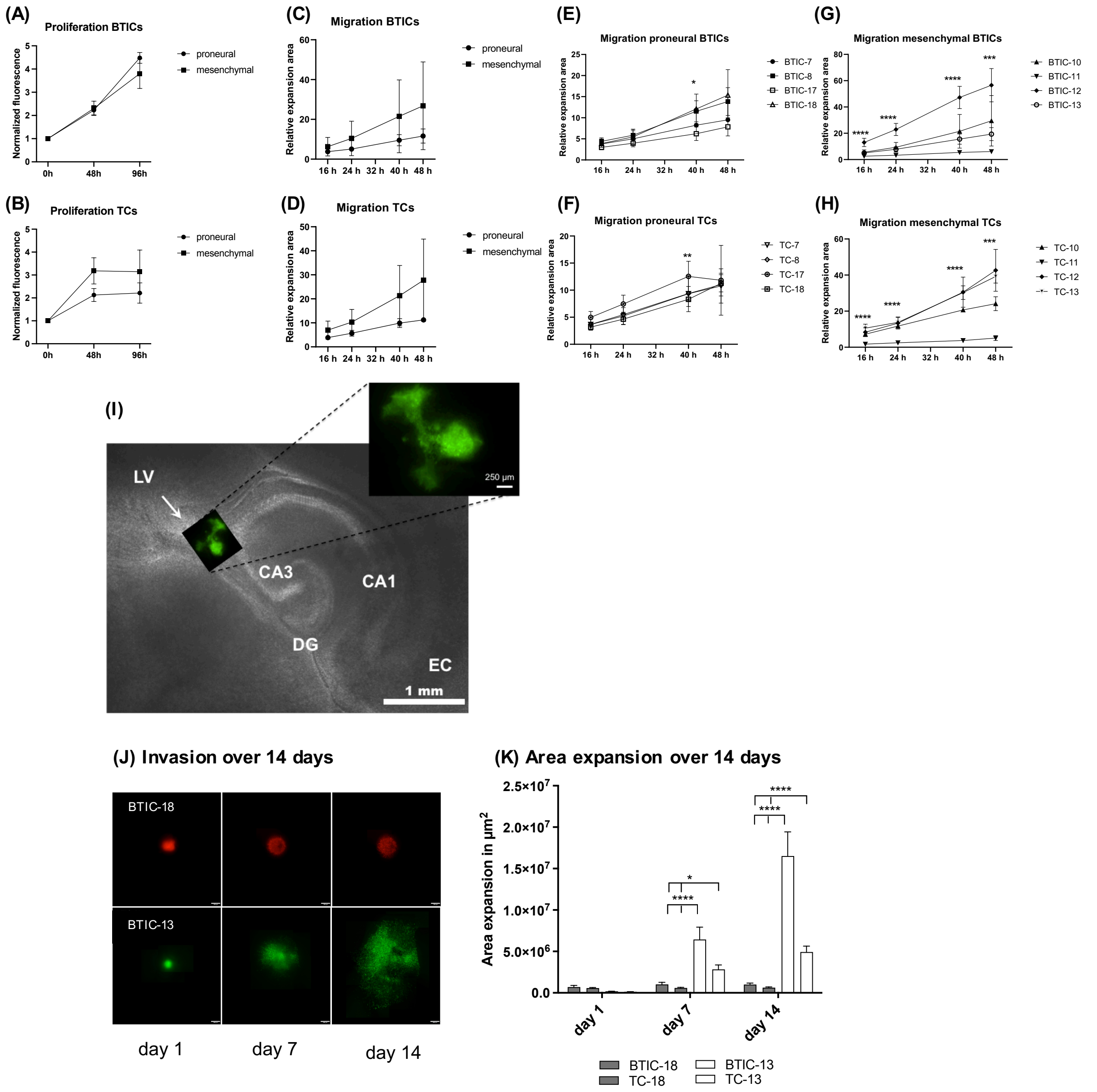

Supplementary Figure S2: Proliferation, migration and invasion of BTICs and TCs. Proliferation of BTICs (proneural BTIC-7, -8, -17, -18 and mesenchymal BTIC-10, -11, -12, -13) and TCs (proneural TC-7, -8, -17, -18 and mesenchymal TC-10, -11, -12, -13) after 48 h and 96 h was determined using the CyQuant Direct Cell Proliferation Assay and normalized to the 0-hour-value (A-B). Migration of proneural and mesenchymal BTICs and TCs (C-H) *in vitro* was assessed using spheroid migration assays and normalized to the 0-hour-value. Furthermore, we monitored invasion on organotypic brain slice cultures (OBSC). Mesenchymal cells were lentivirally transduced with a U57 pHR SFFV GFP plasmid (green), while proneural cells were transduced with pLenti-H1-(shRNA-Neg-control)-Rsv(RFP-Bsd) (red). Two-day-old spheres of these transduced BTICs were transferred onto rat brain slices and inserted in the parahippocampal region (I). Invasion on OBSCs was monitored at 10× magnification under a fluorescent microscope on days 0, 7, and 14. Exemplary images of mesenchymal and proneural BTICs on OBSCs are depicted in (J); the scale bar indicates 250 µm. (K) Mesenchymal BTIC and TC-13 were significantly more invasive than proneural BTIC- and TC-18 on day 14. All assays were performed in triplicate. Asterisks indicate * $p < 0.05$, ** $p < 0.01$, *** $p < 0.001$, and **** $p < 0.0001$.

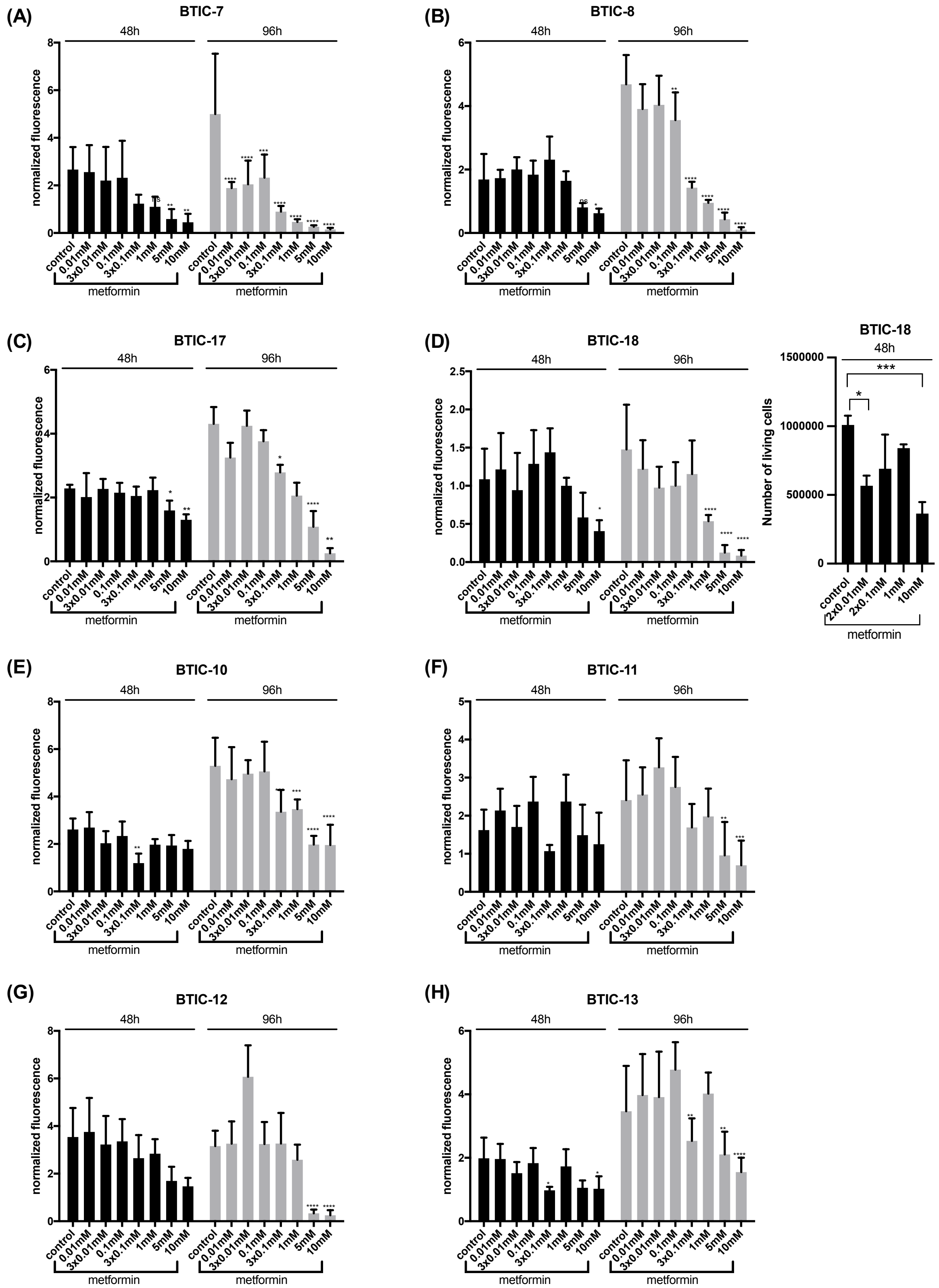
(A)
BTIC-7
(B)
BTIC-8
(C)
BTIC-17
(D)
BTIC-18
(E)
BTIC-10
(F)
BTIC-11
(G)
BTIC-12
(H)
BTIC-13
48h
96h
normalized fluorescence
control
0.01mM
3x0.01mM
0.1mM
3x0.1mM
1mM
5mM
10mM
metformin
Number of living cells
2x0.01mM
2x0.1mM
1500000
1000000
500000

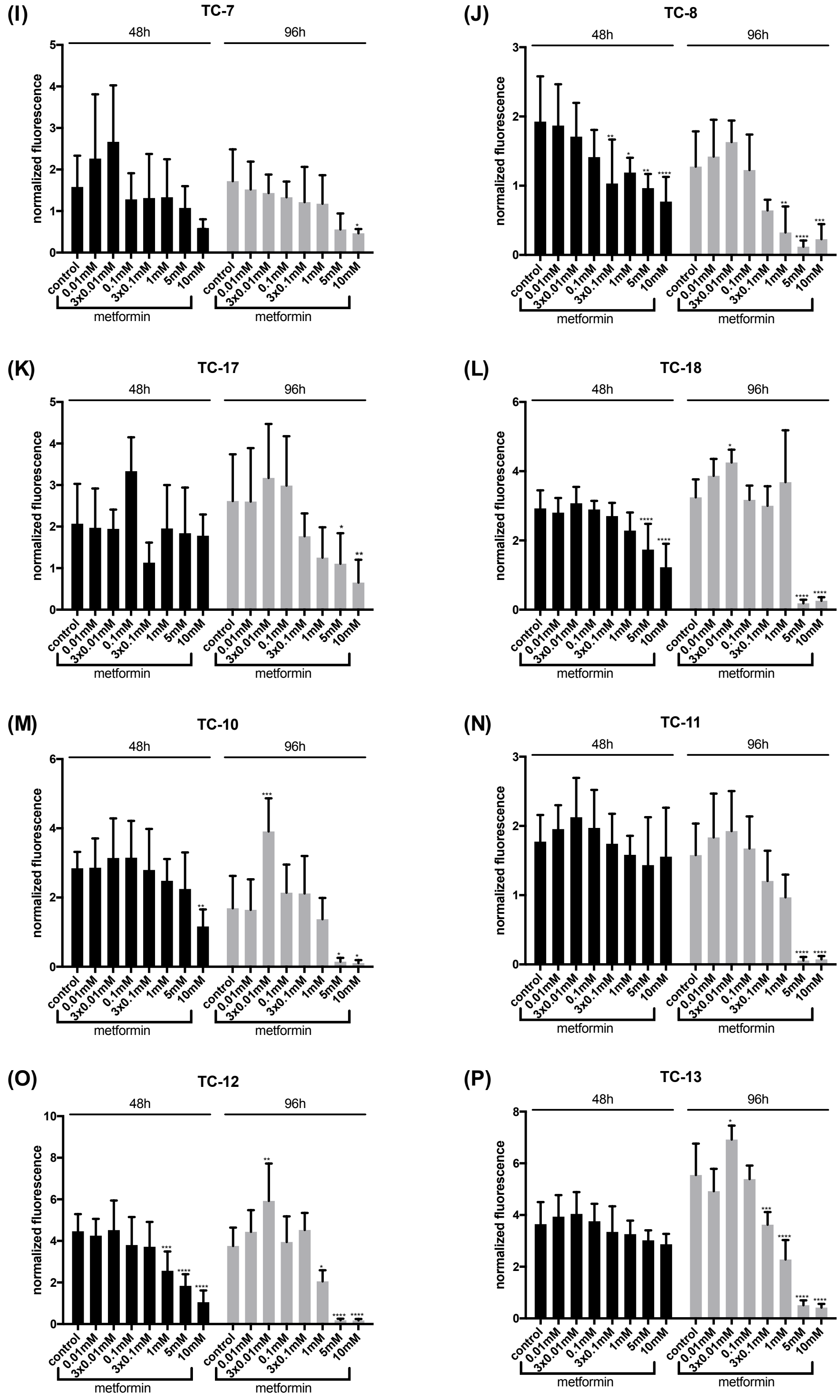

Supplementary Fig. S3: Proliferation of BTICs and TCs under treatment with metformin. Proliferation of proneural BTICs **(A-D)**, mesenchymal BTICs **(E-H)**, proneural TCs **(I-L)** and mesenchymal TCs **(M-P)** after 48 h and 96 h with increasing doses of metformin as indicated was determined using the CyQuant Direct Cell Proliferation Assay and normalized to the 0-hour-value. Proliferation determined by cell count in BTIC-18 after 48h is depicted in D besides the CyQuant results. All assays were performed in triplicate. Asterisks indicate * $p < 0.05$, ** $p < 0.01$, *** $p < 0.001$, and **** $p < 0.0001$.

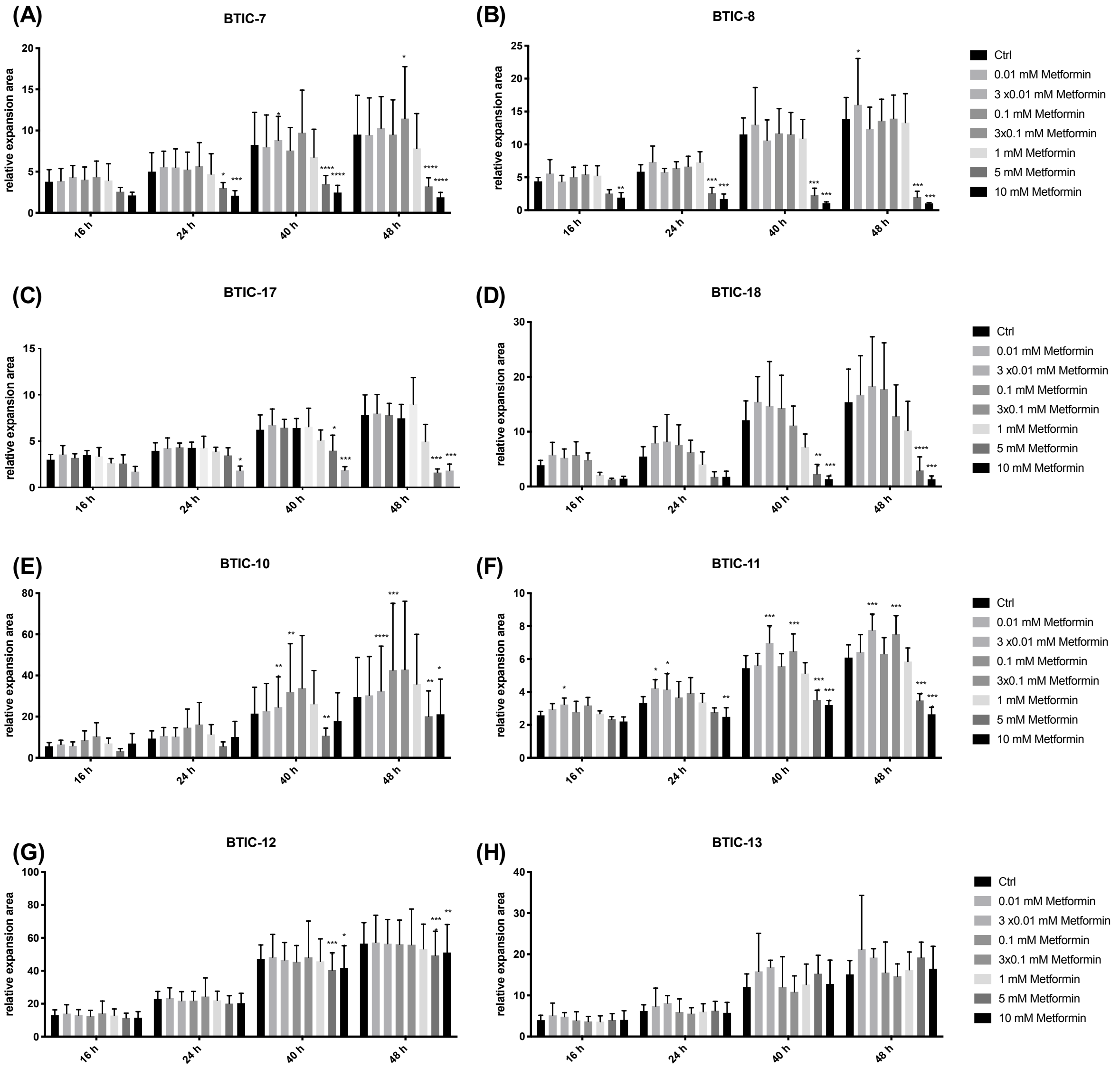

(A)
BTIC-7
(B)
BTIC-8
(C)
BTIC-17
(D)
BTIC-18
(E)
BTIC-10
(F)
BTIC-11
(G)
BTIC-12
(H)
BTIC-13
relative expansion area
16 h
24 h
40 h
48 h
Ctrl
0.01 mM Metformin
3 x0.01 mM Metformin
0.1 mM Metformin
3x0.1 mM Metformin
1 mM Metformin
5 mM Metformin
10 mM Metformin

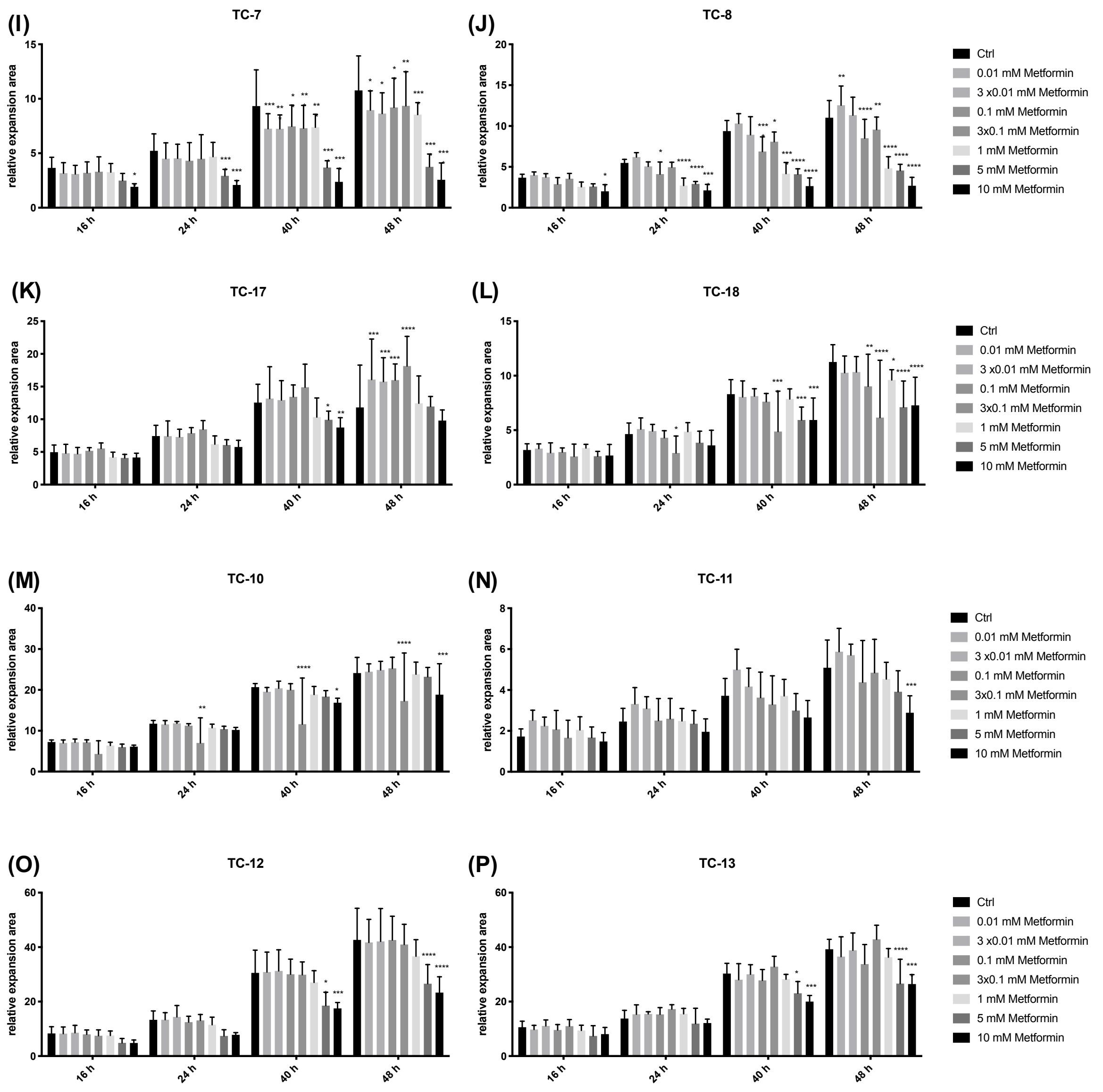

Supplementary Figure S4: Migration of BTICs and TCs under treatment with metformin. Migration of proneural BTICs **(A-D)**, mesenchymal BTICs **(E-H)**, proneural TCs **(I-L)** and mesenchymal TCs **(M-P)** was assessed after 16, 24, 40 and 48 h of treatment with increasing doses of metformin as indicated. Migration was determined using spheroid migration assays and normalized to the 0-hour-value. All assays were performed in triplicate. Asterisks indicate * $p < 0.05$, ** $p < 0.01$, *** $p < 0.001$, and **** $p < 0.0001$.

(A) Relative proliferation at 96h 1mM metformin/control

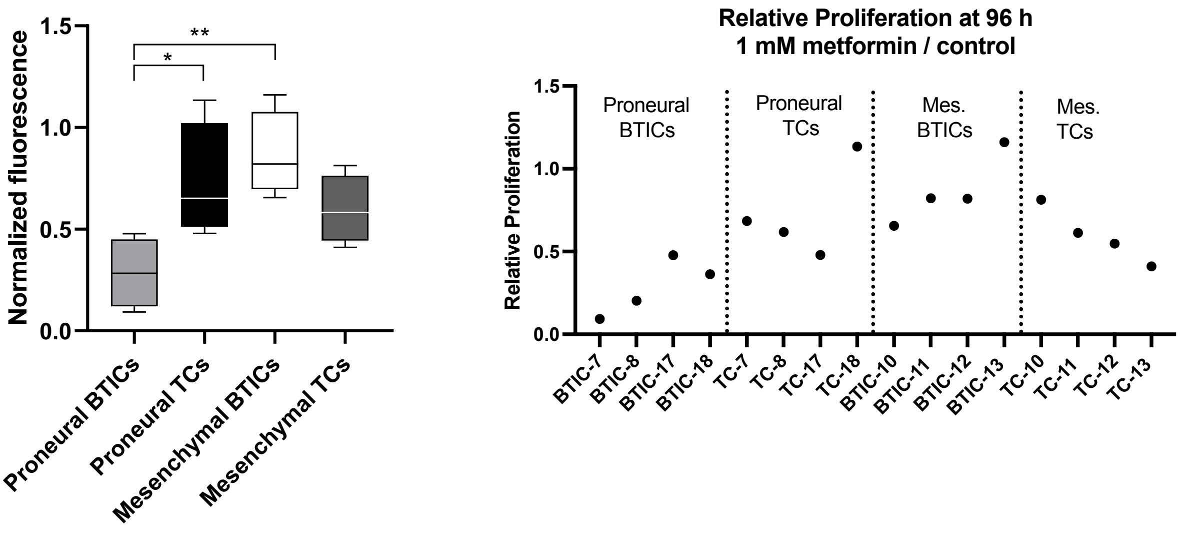

(B) Relative migration at 24h 10mM metformin/control

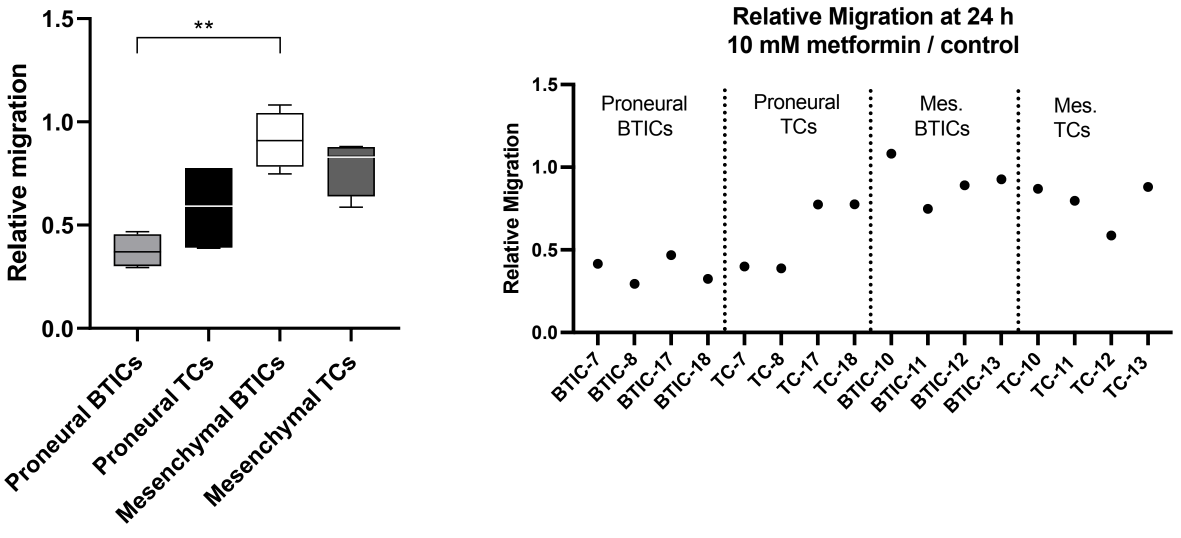

Supplementary Fig. S5: Summary of effects of metformin on proneural and mesenchymal BTICs and TCs. **(A)** We compared proliferation of proneural (-7, -8, -17, -18) and mesenchymal BTICs (10, -11, -12, -13) and corresponding TCs with or without 1 mM metformin after 96 h of treatment. Proliferation was normalized to the 0-hour-value. **(B)** Migration of proneural (-7, -8, -17, -18) and mesenchymal (10, -11, -12, -13) BTICs and TCs with or without 10 mM metformin was measured after 24 h of treatment. Migration was normalized to the 0-hour-value. Asterisks indicate * $p < 0.05$, ** $p < 0.01$.

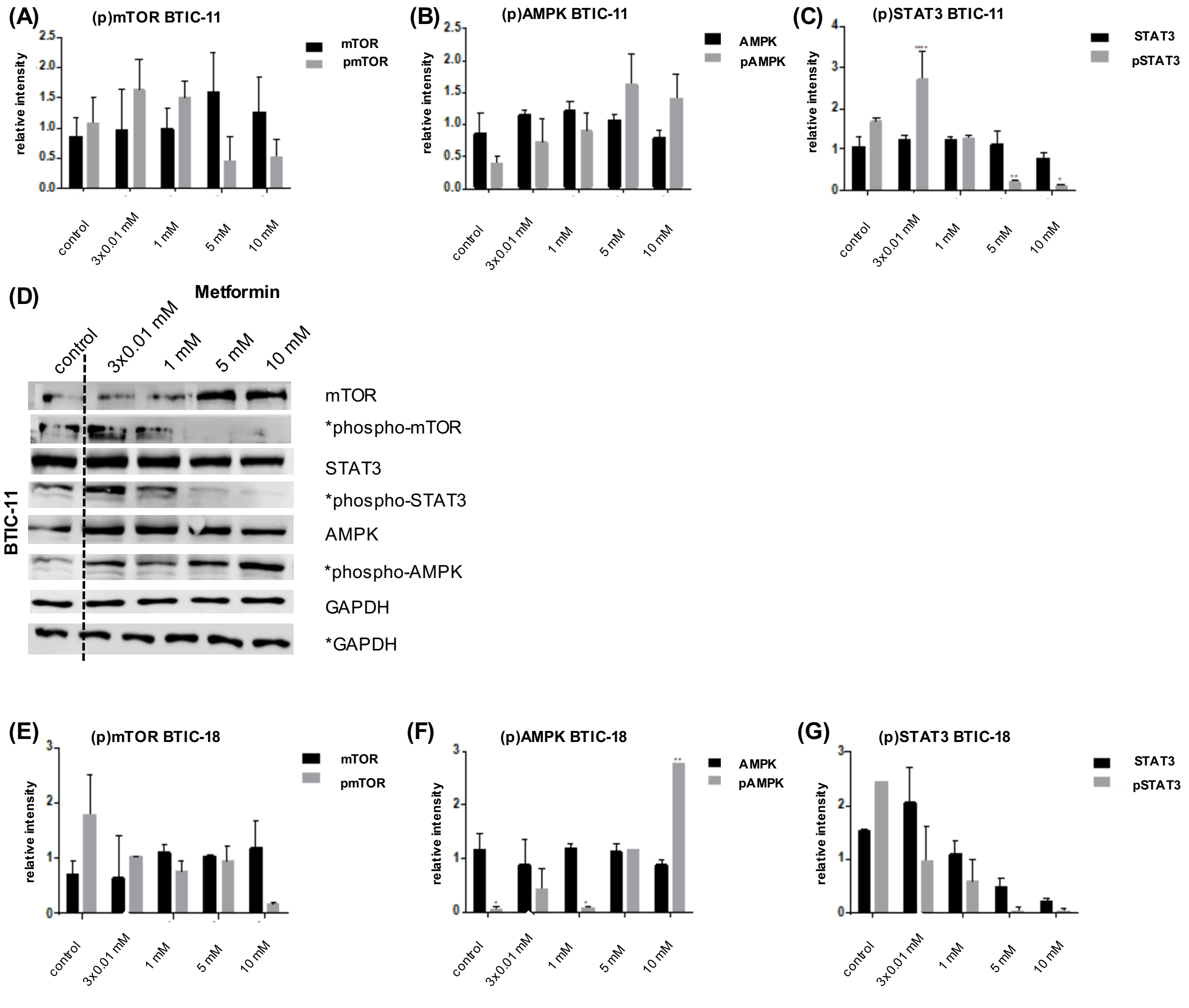

Supplementary Fig. S6: Signaling of BTIC-11 and Western Blot quantification. Protein expression of **(A)** (p)mTOR, **(B)** (p)AMPK and **(C)** (p)STAT3 was explored after 48 h in BTIC-11. One representative Western Blot of BTIC-11 is depicted in **(D)**. Corresponding Western Blot quantifications for BTIC-18 are shown under **(E-G)**. Western blots were repeated three times with at least two biological replicates and quantified using Image J, version 1.49. Asterisks indicate * $p < 0.05$, ** $p < 0.01$.

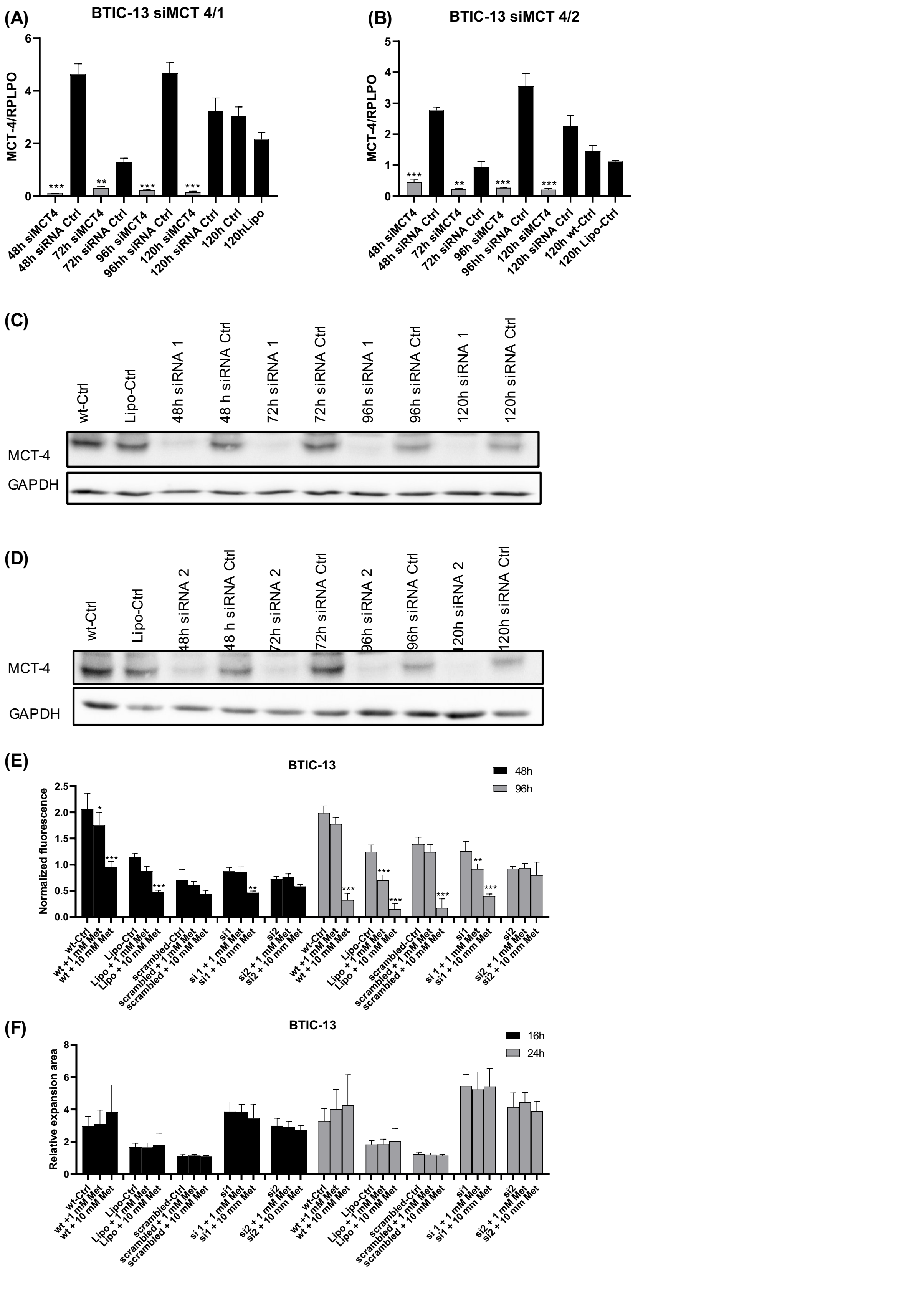

Supplementary Fig. S7. *MCT4* expression and regulation. mRNA expression of *MCT4* after transient transfection with **(A)** siMCT 4/1 and **(B)** siMCT 4/2 was measured using RT-qPCR at the indicated time points in BTIC-13. Transient transfection with **(C)** siMCT 4/1 and **(D)** siMCT 4/2 leads to strong inhibition of MCT4 protein expression over 120 h. **(E)** Treatment with siMCT 4/1 or 4/2 and metformin does not lead to additive effects on cell proliferation as explored by CyQuant Direct Cell Proliferation assays at 48 and 96 h in BTIC-13. **(F)** Treatment with siMCT 4/1 or 4/2 and metformin does not lead to

additive effects on cell migration as explored by spheroid migration assays at 16 and 24 h in BTIC-13. All assays were performed in triplicate. Asterisks indicate * $p < 0.05$, ** $p < 0.01$, *** $p < 0.001$, and **** $p < 0.0001$.

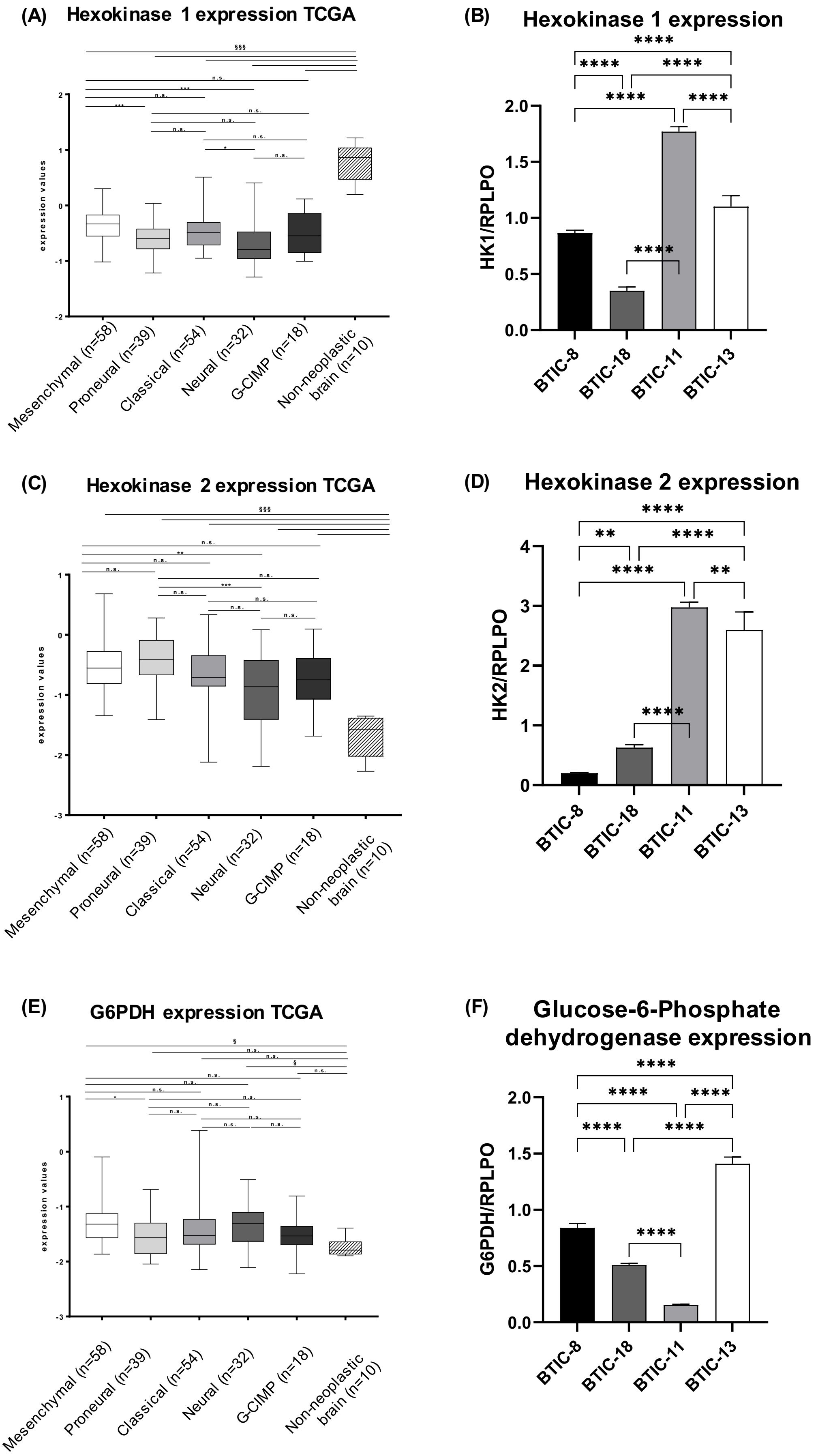
(A)
Hexokinase 1 expression TCGA
§§§
expression values
Mesenchymal (n=58)
Proneural (n=39)
Classical (n=54)
Neural (n=32)
G-CIMP (n=18)
Non-neoplastic brain (n=10)
(B)
Hexokinase 1 expression
HK1/RPLPO
BTIC-8
BTIC-18
BTIC-11
BTIC-13
(C)
Hexokinase 2 expression TCGA
§§§
expression values
Mesenchymal (n=58)
Proneural (n=39)
Classical (n=54)
Neural (n=32)
G-CIMP (n=18)
Non-neoplastic brain (n=10)
(D)
Hexokinase 2 expression
HK2/RPLPO
BTIC-8
BTIC-18
BTIC-11
BTIC-13
(E)
G6PDH expression TCGA
expression values
Mesenchymal (n=58)
Proneural (n=39)
Classical (n=54)
Neural (n=32)
G-CIMP (n=18)
Non-neoplastic brain (n=10)
(F)
Glucose-6-Phosphate dehydrogenase expression
G6PDH/RPLPO
BTIC-8
BTIC-18
BTIC-11
BTIC-13

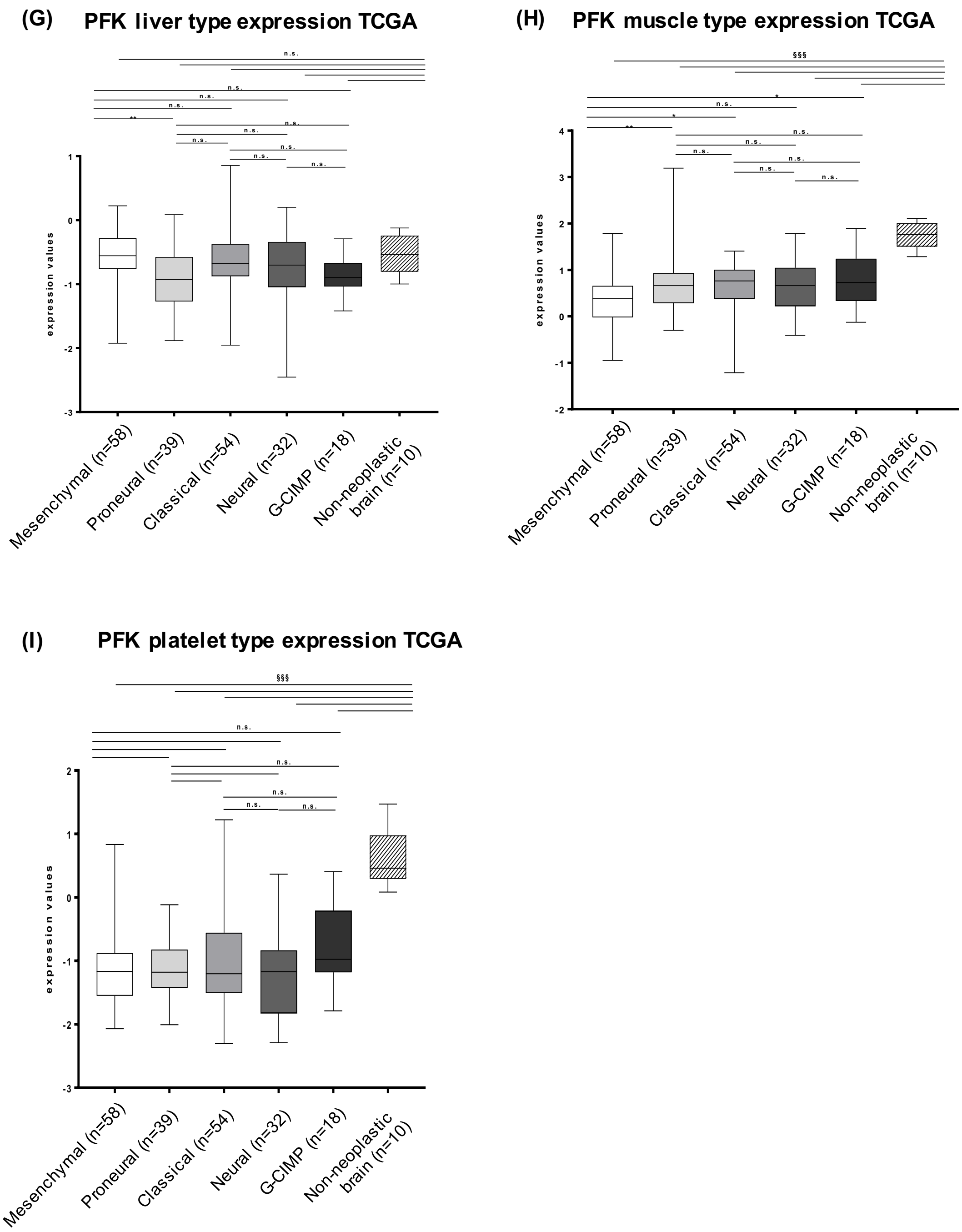

Supplementary Fig. S8. Expression of genes for glycolytic enzymes. Gene expression of hexokinase-1 in TCGA data **(A)** and in BTIC-8, 18, 11, and 13 **(B)**. Expression of hexokinase-2 in TCGA data and in BTIC-8, 18, 11, and 13 is shown under **(C)** and **(D).** Gene expression of *G6PDH* was explored using TCGA data **(E)** and compared to data from BTIC-8, 18, 11, and 13 **(F)**. **(G, H and I)** Expression of liver type, muscle type and platelet type *PFK* in TCGA. Asterisks indicate * $p < 0.05$, ** $p < 0.01$, *** $p < 0.001$, and **** $p < 0.0001$. § indicate $p < 0.05$, §§ indicate $p < 0.01$, §§§ indicate $p<0.001$, and §§§§ indicate $p < 0.0001$ as compared to non-neoplastic brain.

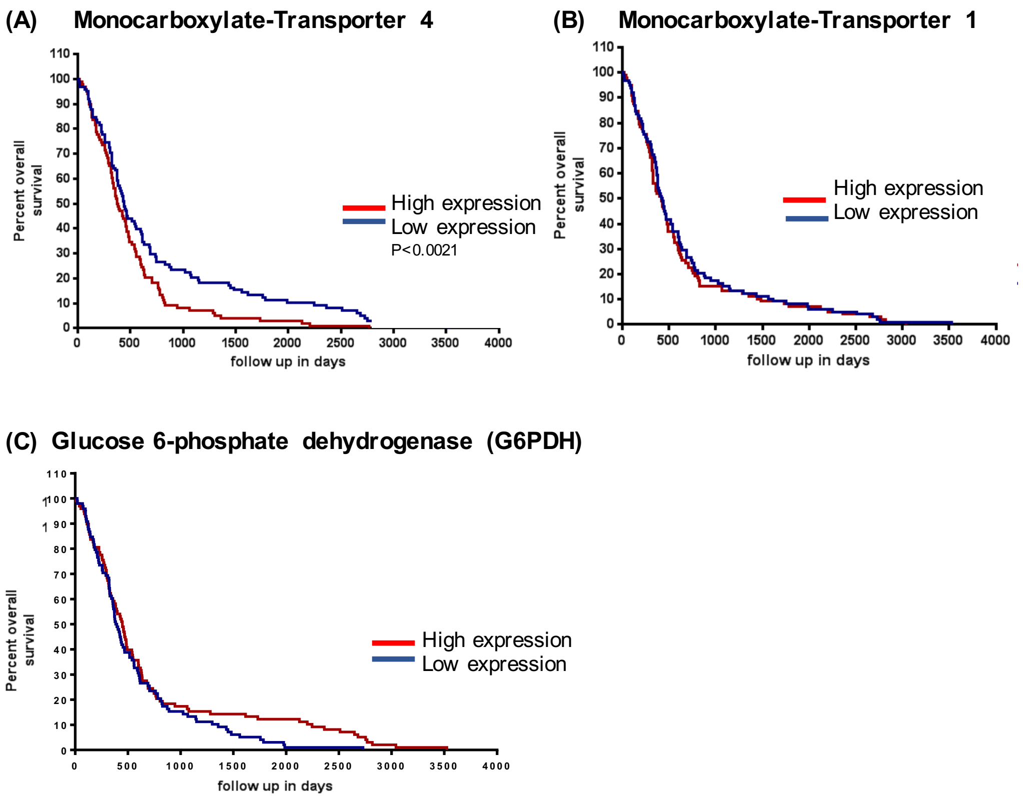

Supplementary Fig. S9: Relation of main results to survival data in TCGA. **(A)** *MCT4*, but not **(B)** *MCT1* gene expression correlated with survival in patients documented in TCGA. **(C)** For *G6PDH* gene expression there was a trend for improved survival among patients with high expression.
