# Supplementary material for: Metabolic Heterogeneity of Brain Tumor Cells of Proneural and Mesenchymal Origin
Source: Int J Mol Sci. 2022 Oct 1;23(19):11629. doi: 10.3390/ijms231911629 (PMC9569970; doi:10.3390/ijms231911629)
Supplement: Supplementary file 1 [file ijms-23-11629-s001.zip › Supplementary Tables.pdf]

Suppl. Table S1. Authentification and characteristics of BTICs.

Authentication of BTIC cell lines was performed at Eurofins Genomics Europe Applied Genomics GmbH (Ebersberg, Germany). Here, DNA isolation was carried out from cell pellet (cell layer). Genetic characteristics were determined by PCR-single-locus-technology. Sixteen independent PCR-systems D8S1179, D21S11, D7S820, CSF1PO, D3S1358, TH01, D13S317, D16S539, D2S1338, AMEL, D5S818, FGA, D19S433, vWA, TPOX and D18S51 were investigated. (ASN-0002 core markers are colored dark grey, Thermo Fisher, AmpFlSTR® Identifiler® Plus PCR Amplification Kit). In parallel, positive and negative controls were carried out yielding correct results.

Regarding MGMT methylation status, it differed between the cell lines (as determined by pyrosequencing), while IDH mutation was negative for all cells in culture. GS=gliosarcoma, GB=glioblastoma, pro.=proneural; mes.=mesenchymal

**Cell line**	**BTIC-8**	**BTIC-11**	**BTIC-13**	**BTIC-18**
**Sample Code**	CL00005293	CL00005295	CL00005297	CL00005299
**D8S1179**	13,15	9,13	10,14	14,14
**D21S11**	30,30	30,32.2	31.2,33.2	31,32.2
**D7S820**	10,11	8,9	10,11	9,9
**CSF1PO**	10,12	11,11	11,12	12,13
**D3S1358**	16,16	17,18	15,18	16,18
**TH01**	6,8	8,8	9.3,9.3	7,9.3
**D13S317**	8,13	9,9	11,11	12,13
**D16S539**	9,11	11,13	8,9	9,12
**D2S1338**	17,24	16,17	21,24	17,19
**D19S433**	13.2,15	14,15.2	14,14	12,12
**vWA**	17,17	16,18	18,19	15,17
**TPOX**	8,9	8,8	8,12	8,11
**D18S51**	12,16	17,17	13,13	12,16
**AMEL**	X,X	X,Y	X,Y	X,Y
**D5S818**	11,11	12,13	11,12	12,12
**FGA**	18,22.2	20,21	19,22	20,23
**Growth *in vitro***	spheres	semi-adherent	adherent	semi-adherent
**Molecular subtype**	Pro.	Mes.	Mes.	Pro.
**MGMT-methylation (%)**	2	12	32	0
**IDH (wt/mut.)**	wt	wt	wt	wt
**$CD133^+$ (%)**	3	6	87	0
**$CD15^+$ (%)**	67	14	1	40
**$A2B5^+$ (%)**	25	5	0	7

Suppl. Table S2. mRNA microarrays with glycolysis as hallmark. Glycolysis was investigated as a hallmark to discriminate proneural and mesenchymal BTICs. Using GSEA, we found glycolytic genes to be differentially expressed between proneural and mesenchymal BTICs es=enrichment score). The strongest enrichment in mesenchymal BTICs was found for the transforming growth factor beta (TGF-beta). Abbreviations: Pro. = proneural; Mes. = mesenchymal.

Gene symbol	Gene title	Rank in gene list	Rank metric score	Running es	Core Enrichment	
B3GAT1	beta-1,3-glucuronyltransferase 1 (glucuronosyltransferase P)	109	20.490.000.247.955.300	-0.031567916	No	Pro.
SDC3	syndecan 3 (N-syndecan)	330	10.670.000.314.712.500	-0.18104587	No	Pro.
EFNA3	ephrin-A3	383	0.963999987	-0.19648324	No	Pro.
ME2	malic enzyme 2, NAD(+)-dependent, mitochondrial	416	0.866999984	-0.19833168	No	Pro.
KIF2A	kinesin heavy chain member 2A	534	0.545000017	-0.27845424	No	Pro.
HDLBP	high density lipoprotein binding protein (vigilin)	557	-0.330000013	-0.2871611	No	Mes.
CLDN3	claudin 3	591	-0.552999973	-0.2986004	No	Mes.
SLC16A3	solute carrier family 16, member 3 (monocarboxylic acid transporter 4)	713	-0.838	-0.37379402	No	Mes.
SPAG4	sperm associated antigen 4	719	-0.851999998	-0.3540568	No	Mes.
null	null	727	-0.885999978	-0.33499932	No	Mes.
G6PD	glucose-6-phosphate dehydrogenase	782	-1.059.000.015.258.780	-0.34941158	No	Mes.
GLRX	glutaredoxin (thioltransferase)	899	-1.440.999.984.741.210	-0.4036772	Yes	Mes.
COL5A1	collagen, type V, alpha 1	956	-1.656.000.018.119.810	-0.40303412	Yes	Mes.
IL13RA1	interleukin 13 receptor, alpha 1	971	-17.070.000.171.661.300	-0.36673582	Yes	Mes.
CD44	CD44 molecule (Indian blood group)	983	-1.75	-0.32679078	Yes	Mes.
P4HA2	procollagen-proline, 2-oxoglutarate 4-dioxygenase (proline 4-hydroxylase), alpha polypeptide II	1076	-21.579.999.923.706.000	-0.34145737	Yes	Mes.
IER3	immediate early response 3	1124	-23.499.999.046.325.600	-0.314083	Yes	Mes.
B4GALT1	UDP-Gal:betaGlcNAc beta 1,4-galactosyltransferase, polypeptide 1	1128	-2.384.999.990.463.250	-0.24987064	Yes	Mes.

TPBG	trophoblast glycoprotein	1223	-3.431.999.921.798.700	-0.23056073	Yes	Mes.
MET	met proto-oncogene (hepatocyte growth factor receptor)	1237	-3.812.000.036.239.620	-0.1346157	Yes	Mes.
TGFBI	transforming growth factor, beta-induced, 68kDa	1247	-5.078.999.996.185.300	1.61E-01	Yes	Mes.
